# Supplementary figures and images for: Zipime-Weka-Schista study protocol: a longitudinal cohort study and economic evaluation of an integrated home-based approach for genital multipathogen screening in women, including female genital schistosomiasis, human papillomavirus, Trichomonas and HIV in Zambia
Source: BMJ Open. 2024 Jun 10;14(6):e080395. doi: 10.1136/bmjopen-2023-080395 (PMC11168163; doi:10.1136/bmjopen-2023-080395)

**Supplementary figure 1: 3 D models for the teaching of self-sampling**

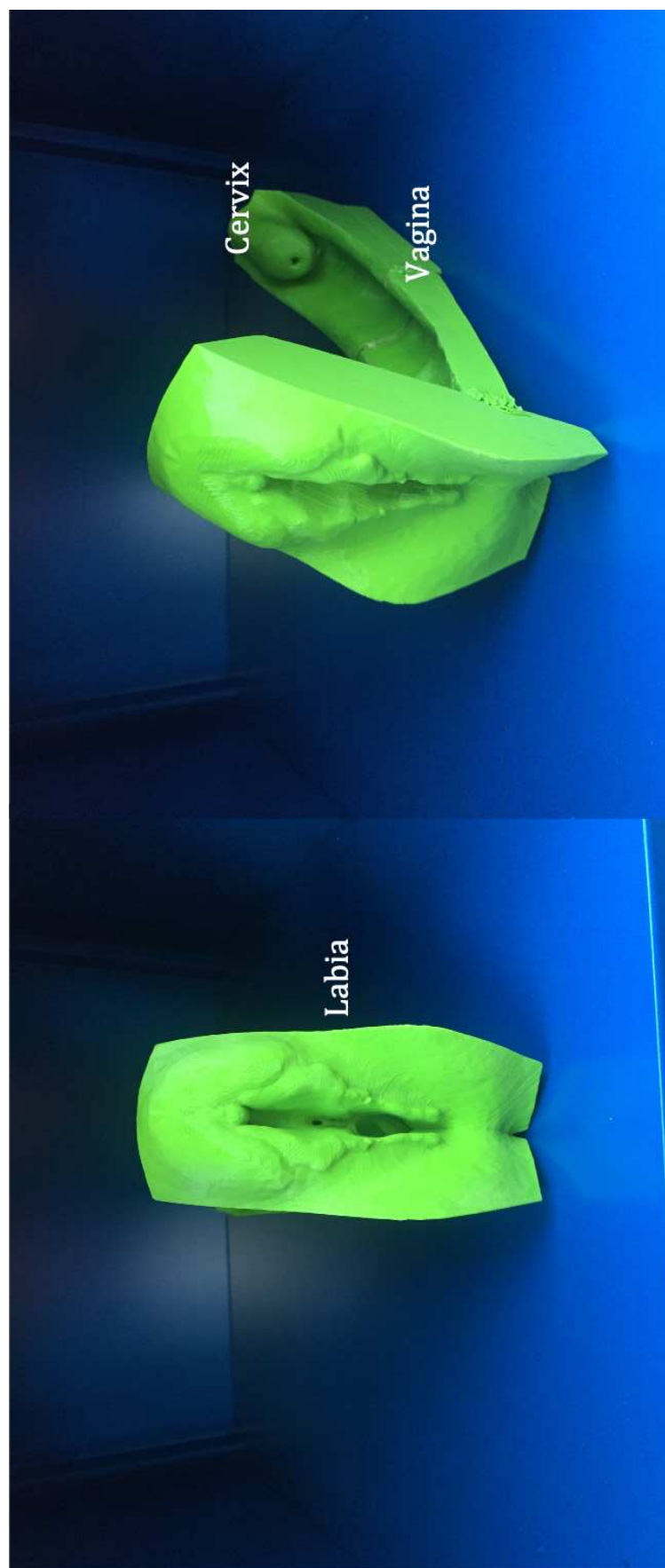

Supplement: Supplementary data [file bmjopen-2023-080395supp001.pdf]
