# Supplementary material for: Zipime-Weka-Schista study protocol: a longitudinal cohort study and economic evaluation of an integrated home-based approach for genital multipathogen screening in women, including female genital schistosomiasis, human papillomavirus, Trichomonas and HIV in Zambia
Source: BMJ Open. 2024 Jun 10;14(6):e080395. doi: 10.1136/bmjopen-2023-080395 (PMC11168163; doi:10.1136/bmjopen-2023-080395)
Supplement: Author's manuscript [file bmjopen-2023-080395.draft_revisions.pdf]

BMJ Open is committed to open peer review. As part of this commitment we make the peer review history of every article we publish publicly available.

When an article is published we post the peer reviewers' comments and the authors' responses online. We also post the versions of the paper that were used during peer review. These are the versions that the peer review comments apply to.

The versions of the paper that follow are the versions that were submitted during the peer review process. They are not the versions of record or the final published versions. They should not be cited or distributed as the published version of this manuscript.

BMJ Open is an open access journal and the full, final, typeset and author-corrected version of record of the manuscript is available on our site with no access controls, subscription charges or pay-per-view fees (http://bmjopen.bmj.com).

If you have any questions on BMJ Open's open peer review process please email info.bmjopen@bmj.com

BMJ Open

The Zipime-Weka-Schista study protocol: a longitudinal cohort study of an integrated home-based approach for genital multi-pathogen screening in women, including female genital schistosomiasis, HPV, Trichomonas and HIV in Zambia

Journal:	*BMJ Open*
Manuscript ID	bmjopen-2023-080395
Article Type:	Protocol
Date Submitted by the Author:	29-Sep-2023

Complete List of Authors:	Shanaube, Kwame; Zambart<br>Ndubani, Rhoda; Zambart<br>Kelly, Helen; London School of Hygiene and Tropical Medicine, Clinical Research Department<br>Webb, Emily; London School of Hygiene and Tropical Medicine, Faculty of Infectious and Tropical Diseases<br>Mayaud, Philippe; London School of Hygiene & Tropical Medicine Faculty of Infectious and Tropical Diseases, Clinical Research<br>Lamberti, Olimpia; London School of Hygiene and Tropical Medicine, Clinical Research Department<br>Fitzpatrick, Jennifer; Zambart<br>Kasese, Nkatya; Zambart<br>Sturt, Amy; Veterans Affairs Health Care System, Department of Infectious Diseases<br>Van Lieshout, Lisette; Leiden University Medical Center<br>Van Dam, Govert; Leiden University Medical Center<br>Corstjens, Paul; Leiden University Medical Center, Department of Cell and Chemical Biology<br>Kosloff, Barry; Zambart<br>Bond, Virginia; London School of Hygiene & Tropical Medicine Centre of Global Change and Health<br>Hayes, Richard; London School of Hygiene and Tropical Medicine, Epidemiology and Population Health<br>Terris-Prestholt, Fern; London School of Hygiene & Tropical Medicine<br>Webster, Bonnie; Natural History Museum<br>Vwalika, Bellington; University of Zambia School of Medicine, Department of Obstetrics & Gynaecology<br>Hansingo, Isaiah; Livingstone Central Hospital, Gynecology<br>Ayles, Helen; London School of Hygiene & Tropical Medicine; Zambart<br>Bustinduy, Amaya; London School of Hygiene and Tropical Medicine, Clinical Research; London School of Hygiene & Tropical Medicine

Keywords:	Adolescent, PUBLIC HEALTH, SEXUAL MEDICINE, Molecular diagnostics < INFECTIOUS DISEASES, PARASITOLOGY, Colposcopy < GYNAECOLOGY

For peer review only - http://bmjopen.bmj.com/site/about/guidelines.xhtml

The Zipime-Weka-Schista study protocol: a longitudinal cohort study of an integrated home-based approach for genital multi-pathogen screening in women, including female genital schistosomiasis, HPV, Trichomonas and HIV in Zambia

*Kwame Shanaube[1], Rhoda Ndubani[1], Helen Kelly[2,] Emily L. Webb[3], Philippe Mayaud[2], Olimpia Lamberti[2], Jennifer Fitzpatrick[1], Nkatya Kasese[1], Amy Sturt[5], Lisette van Lieshout[6], Govert J. van Dam[6], Paul L.A.M. Corstjens[7], Barry Kosloff[1], Virginia Bond[1,2], Richard Hayes[3], Fern Terris-Prestholt[8,9] Bonnie Webster[10], Bellington Vwalika[11], Isaiah Hansingo[4] , Helen Ayles[1,2], Amaya L. Bustinduy[2]

1. Zambart, Lusaka, Zambia
2. Department of Clinical Research, London School of Hygiene & Tropical Medicine, Keppel Street, London WC1E 7HT, United Kingdom
3. Department of Infectious Disease Epidemiology, London School of Hygiene & Tropical Medicine, Keppel Street, London, United Kingdom
4. Livingstone Central Hospital, Livingstone, Zambia
5. Department of Infectious Diseases, Veterans Affairs Health Care System, California, USA
6. Department of Parasitology, Leiden University Medical Center, Leiden, The Netherlands
7. Department of Cell and Chemical Biology, Leiden University Medical Center, Leiden, The Netherlands.
8. Department of Global Health and Development, London School of Hygiene & Tropical Medicine, Keppel Street, London, United Kingdom
9. UNAIDS, WHO, Geneva, Switzerland
10. Wolfson Wellcome Biomedical Laboratories, Department of Zoology, Natural History Museum, Cromwell Road, London, United Kingdom
11. Department of Gynecology, University of Zambia, Lusaka, Zambia

***Corresponding author:**

Prof. Amaya Bustinduy

London School of Hygiene & Tropical Medicine

Keppel Street

WC1E 7HT- London, UK

Amaya.Bustinduy@lshtm.ac.uk

Word count: 3955

Figures:2

Tables:1

For peer review only

ABSTRACT

Introduction

Multiplathogen home-based self-sampling offers an opportunity to increase access to screening and treatment. Infections include sexually transmitted (HIV, *Trichomonas vaginalis (Tv),* human papillomavirus [HPV] for cervical precancer detection) and non-sexually transmitted pathogens (*Schistosoma haematobium (Sh))* pathogens, in endemic settings with high co-infection prevalence. Chronic infection may lead to disability (female genital schistosomiasis (FGS) and death (cervical cancer). The Zipime-Weka-Schista (Do self-testing sister!) aims to evaluate the validity, acceptability, uptake, impact and cost-effectiveness of *multi-pathogen* self-sampling for genital infections among women in Zambia.

Methods and Analysis

This is a longitudinal cohort study aiming to enrol 2,500 non-pregnant, sexually active and non-menstruating women aged 15-50 years from two districts in Zambia with two year follow up. During home visits, community health workers offer HIV and *Tv* self-testing and cervicovaginal self-swabs for (i) HPV by GeneXpert and, (ii) *Sh* DNA detection by conventional (PCR) and isothermal (RPA) molecular methods. *Schistosoma* ova and circulating anodic antigen are detected in urine. At a clinic follow-up midwifes perform same procedures and obtain hand-held colposcopic images. High-risk -HPV positive women are referred for a two-quadrant cervical biopsy according to age and HIV status. A *cost-effectiveness* analysis is conducted in parallel.

Ethics and dissemination

The University of Zambia Biomedical Research Ethics Committee (UNZABREC) approved the study in September 2021 (reference: 1858-2021), the London School of Hygiene and Tropical Medicine(LSHTM) (reference: 25258) and Ministry of Health and local superintendents. Written informed consent was obtained from all participants prior to enrolment. Data will be available upon request on LSHTM Data Compass. Identifiable data collected is stored securely and their confidentiality protected in accordance with the Data Protection Act 1998.

Keywords: Female genital schistosomiasis, *Schistosoma haematobium*, HIV, *Trichomonas vaginalis*, human papillomavirus (HPV), cervical cancer, self-sampling, acceptability, cost-effectiveness, hand-held colposcopy, RPA

For peer review only

For peer review only - http://bmjopen.bmj.com/site/about/guidelines.xhtml

Strenghts and Limitations

- First longitudinal cohort study of female genital schistosomiasis (FGS)
- Home 'package' testing for validation and acceptability of self-sampling (FGS, HR-HPV) and self-testing (HIV and Trichomonas)
- First study addressing *cost-effectiveness* of community-based integration of FGS within the wider sexual and reproductive health screening in Zambia
- Existing sexually transmitted infection related stigma in the communities may difficult recruitment
- Limited availability of histopathology laboratories may delay results

For peer review only

For peer review only - http://bmjopen.bmj.com/site/about/guidelines.xhtml

INTRODUCTION

It is estimated that around 45 million women living in sub-Sahara Africa (SSA) are affected by Female genital schistosomiasis (FGS), a chronic, neglected and disabling gynaecological disorder associated with infertility, dyspareunia and symptoms mimicking sexually transmitted infections (STIs) [1 2]. FGS is difficult to diagnose and is part of the wider spectrum of urogenital disease caused by the waterborne parasite *Schistosoma haematobium* (*Sh*). In Zambia, reported FGS prevalence ranges from 33-75% of those with urinary schistosomiasis in endemic areas, suggesting a potentially high FGS burden. Data is not yet available on the FGS burden in the general population of Zambia. Furthermore, awareness of the disease and *non-sexual* mode of transmission is largely absent in endemic communities where information and education are urgently needed [3].

Conventional FGS diagnosis is challenging as it relies on costly equipment and high-level specialized training is seldom available in resource-limited countries. Accurate estimation of disease burden is therefore hindered. However, the past decade has seen a dramatic increase in knowledge generation on many facets of FGS, and particularly on diagnostic strategies closer to the point-of-care [4]. Studies have shown acceptable sensitivity of genital swabs for the DNA detection of *Sh* from women's genital tract, potentially offering, for the first time, a scalable diagnostic approach for a parasitic disease that has been unacceptably neglected [3 4]. Genital self-swabs are well accepted by participants and are known to increase uptake across settings [5 6].

There is growing evidence of an association of FGS with prevalent HIV, and suggestion of increased incidence and transmission of HIV in the presence of schistosome infections in women [7]. The lesions and tissue damage caused by FGS can provide an easy route for STI infections. [8]. Furthermore, women with schistosome worm infections and with FGS are potentially far more susceptible and at an increased risk of becoming HIV positive [9]. However, there is still a knowledge vacuum on the role of STIs in HIV acquisition in the presence of FGS, despite both STIs and *S.haematobium* infection being shown to be independently associated with HIV acquisition.[8 10 11].

For peer review only - http://bmjopen.bmj.com/site/about/guidelines.xhtml

There have been small studies suggesting an increased prevalence of high risk (HR-) HP, the etiological agent of cervical cancer, in women with FGS compared to those without [12] and higher odds of having an abnormal visual examination with acetic acid [VIA][13] and some suggestion of potential links between FGS and cervical cancer [14 15]. In Zambia, cervical cancer is the most common female cancer, particularly in women aged 15 to 44 years[16 17] and with high associated mortality (43.4 per 100,000 women). The lack of accessible screening and treatment programs [18] and HPV vaccination contribute to the high cervical cancer incidence in these settings[19] HPV-DNA based tests are recommended for primary screening of cervical cancer as part of the WHO 2021 cervical cancer screening and treatment guidelines [20], as better strategies than visual diagnosis. [21 22]. Despite validated self-sampling strategies for the detection of oncogenic or HR-HPV genotypes, community-based screening is not yet widely adopted alone or integrated with screening for other pathogens due to the high cost and infrastructure requirements of currently available tests [23].

This study protocol describes the development of a community-based comprehensive package for SRH screening in Zambia, including *sexually* (HPV, HIV, *Tv*) and *non-sexually* transmitted infections (*Sh)* in different endemicity settings with the long-term aim of preventing life threatening and disabling conditions such as cervical cancer, HIV and FGS. The overall justification of this study lies in the dearth of research exploring the feasibility of integrated screening and testing strategies of genital co-infections, and the epidemiological associations between them.[24-26].

METHODS AND DESIGN

Study design

The Zipime Weka Schista (Do self-testing sister!) study is a longitudinal cohort designed as a holistic approach to FGS detection at scale in the community. The study design will test the hypothesis that integration of home self-sampling for the screening of FGS in conjunction with HR-HPV and self-testing for HIV and STIs is an *diagnostically accurate*, *cost-effective* and *self-empowering* strategy that will increase the detection of cases and improve access to care for women of reproductive age in SSA.

The two main aims of the Schista study are:

For peer review only - http://bmjopen.bmj.com/site/about/guidelines.xhtml

1. To assess the performance of genital self-sampling, compared to healthcare provider sampling obtained for the detection of *Sh* by molecular testing (real-time PCR and Recombinase Polymerase Assay (RPA)) across two different *Sh* endemicity settings in Zambia and
2. To assess the observed costs of integrating community based multiple pathogen genital self-sampling (*Sh*, HPV, STIs) and oral self-test (HIV) into one-stop home intervention and. project costs at scale.

Our secondary aims are to explore the epidemiological and immunological interrelationships between FGS and HPV as a potential risk factor for cervical cancer and expand knowledge on HIV acquisition in the presence of co- infections. We will also measure *acceptability* and *feasibility* of home-based multiple genital self-sampling and to measure HIV linkage to care and cervical cancer management after home self-sampling and testing. In addition, we will aim to evaluate the effect of FGS and other STI on the diagnostic accuracy of visual inspection for detection of cervical precancer (CIN2+, CIN3+) and to describe the association of FGS, HPV, HIV and STIs with the presence concentration, diversity, and relative abundance of other key cervicovaginal microbiota and infections. Further aims will aim to consolidate testing for FGS and HPV into one field-applicable multiplex molecular assay and to recruit a sub-set of male household members of recruited participants screening male genital schistosomiasis.

Study setting and population

The study is being conducted in Lusaka and Southern provinces of Zambia. The specific study study areas are two sites in Kafue district with high (estimated *Sh* prevalence >70%) and moderate (estimated *Sh* prevalence >50%) prevalence and another site in Livingstone district with low prevalence (estimated *Sh* prevalence <10%). Each woman will have one home visit and one clinic visit at baseline with annual follow up for 2 years. Enrolment of eligible women began in January 2022 and is expected to complete in January 2024.

Inclusion/Exclusion criteria

All non-pregnant, sexually active women aged 15-50 years who are currently not menstruating and are able to provide consent are invited to take part in the study. In addition, women should be Zambian

For peer review only

For peer review only - http://bmjopen.bmj.com/site/about/guidelines.xhtml

residents of the endemic study area for at least one year.Women are excluded prior to their sexual debut ,pregnant or currently menstruating.

Sampling strategy and data collection procedures

Community-based cluster sampling was used to obtain a random sample of participants from the study communities. Communities and their households were mapped and each community was subdivided into blocks of approximately 50 households; each one would yield approximately 30 enrolled women. These community blocks were the primary sampling units, with all households in a selected block visited, and all eligible women aged 15-50 years invited to take part. Blocks were randomly selected using random number generation in Stata, and are being visited sequentially in the order that they were randomly selected. Participating women are assigned a unique identifier. Field data are entered using the London School of Hygiene and Tropical Medicine (LSHTM) ODK system and uploaded to a secure server in Zambia. The data are then transferred to a LSHTM secure server. The Chief Investigator is responsible for the data analysis with the support of the Department of Infectious Diseases Epidemiology at LSHTM. A data management plan is outlined in *Additional file 2.*

Sample size and study power

A total of 2,500 women will be randomly selected from two districts (1,250 from each district). Based on previous assessments of FGS using PCR testing of genital samples from women in the study setting, we assume that 5-10% of genital samples from healthcare provider sampling will have *Sh* detected by molecular testing (5% in the lower endemicity area, 10% in the higher endemicity area). Taking clinic-based sampling as the gold standard, allowing for 10% loss to follow-up between the home and clinic sampling, and assuming that the diagnostic sensitivity and specificity of self-sampling compared to clinic-based sampling for the detection of *Sh* are 70% and 85%, respectively, we will be able to estimate the assumed target diagnostic sensitivity of 70% with a 95% CI of 62.3-76.6% overall, and the assumed target diagnostic specificity of 85% with a 95% CI of 83.4-86.5%. Considering individual districts, we will be able to estimate sensitivity of 70% with a 95% CI of 60.6-78.2% in the higher endemicity area and a 95% CI of 55.9-81.2% in the lower endemicity area. Corresponding confidence intervals for specificity would be 83-87% in each area.

For peer review only - http://bmjopen.bmj.com/site/about/guidelines.xhtml

FIELD PROCEDURES

Community sensitization activities take place to inform target communities of the study aims and promote voluntary participation. Study community workers (SCWs) register expressions of interest and conduct home visits within the randomized zones to women that have expressed interest in participating in the study. All SCWs are females. Every participant has a total of three visits during the study: baseline, 12, and 24 months from recruitment. A schematic study flow is shown in **Figure 1**.

Home visits

Home visits are conducted by SCWs who perform a demonstration of self-sampling, using a 3D model for illustration (supplementary figure 1). SCWs also obtain consent from participants and administer demographic and exposure questionnaires. After obtaining informed consent and completing questionnaires on demography, clinical symptoms and other relevant health and water contact information, as well as a Likert scale for acceptability [27], the SCWs offer partcipants one genital self-swabs is stored in Primestore (Longhorn Vaccines and Diagnostics LLC) for the detection of *Sh* by molecular methods (PCR and RPA) and the second genital swab is stored in Cytopreserve® for HR-HPV detection by Xpert. Participants are also offered two commercial self-tests: one oral (saliva) for HIV [28] and one vaginal self-swab for *Trichomonas vaginalis* (*Tv*) [29]. For both self-tests, results are read by the SCW and given immediately. A confirmatory test is performed on site if there is a positive self-test for HIV. All HIV-positive women are referred to the local clinic for further management and linkage to care. For *Tv*, women are referred for treatment at the clinic visit. A urine sample is analysed via microscopy for *Sh* ova detection and circulating anodic antigen (CAA)[30].

<< Insert Figure 1 here>>

Clinic visit

A follow up visit takes place at a local clinic. A trained healthcare provider (midwife) collects two cervicovaginal swabs and 5 ml of cervicovaginal lavage (CVL). Swabs are placed in Primestore (Longhorn Vaccine and Diagnostics LLC) and CVL are stored at -20 C on site for future detection of : 1) *Sh* DNA by real-

For peer review only - http://bmjopen.bmj.com/site/about/guidelines.xhtml

time PCR as the reference assay for FGS [31]; 2) *Sh* DNA by Xpert® CT/NG (Chlamydia Trachomatis/Neisseria gonorrhoeae) [32] and studies of biomarkers and cervivovaginal microbiome. Approximately 5 ml of blood is collected by venipuncture in an EDTA tube for the detection of *Schistosoma* and STI serology. A hand-held colposcope, with an enhanced visual assessment (EVA) system is used to image the participant's cervix and vagina (MobileODT, Tel Aviv, Israel).

Praziquantel and metronidazole are available free of charge for study partcipants with any positive result[33].

Biopsy procedure

Women testing positive for any *high-risk HPV* by PCR (Xpert® HPV) during the home visit and depending on their age and HIV status *(see below)* are referred for colposcopy. Visual inspection using acetic acid and lugol's iodine *(VIA/VILI)* is performed by a trained gynecologist before biopsy of visible lesions. If no lesions are visible, two random biopsies are taken from normal areas including the transformation zone (at 12 and 6 o'clock), **Figure 2.** Cervical biopsies will be processed at the pathology laboratory at National Health laboratory Service, Johannesburg, South Africa and read using the 2-tier CIN classification system.

<< Insert Figure 2 here>>

HIV-negative women under the age of 25 and women living with HIV (WLHIV) under the age of 20 years have all procedures performed, including HPV testing in the community, but *they do not undergo biopsy in clinic* despite positive HR-HPV results [34]. This strategy follows ethical concerns of unnecessary biopsy and management of cervical lesions based on known HPV clearance and low risk of cervical precancer in young women, [35]. For these women, there is annual longitudinal follow up for two years with repeated infection and clinical re-assessments.

Women diagnosed with cervical precancer and invasive cervical cancer:. If found to have histopathology verified CIN2+, participants are recalled for CIN2+ management. This visit is scheduled at the earliest convenient date once the result is known . In Zambia, all cancer patients are referred to the Cancer Diseases Hospital (CDH) based in Lusaka. Patients are filtered through the district and provincial hospitals serving as primary centres for cervical cancer screening countrywide before being referred to

For peer review only - http://bmjopen.bmj.com/site/about/guidelines.xhtml

CDH for definitive treatment. Treatment at CDH is done according to the nationally and globally developed guidelines [36].

LABORATORY PROCEDURES

The collection, storage and processing of all study samples is summarised in **Table 1**. Urine samples are processed for; 1) ***Urine parasitology*** Approximately 10mL (minimum) of urine is processed by centrifugation to detect *Sh* eggs by microscopy performed at the local government clinics in Kafue and Livingstone in Zambia by a trained technician and 2). ***CAA antigen assay.*** 2 mL of urine are stored at 4 ^{o}C and shipped to Lusaka and further to Leiden University Medical Center, The Netherlands [37].

All genital samples (home/clinic) are processed for; 1) ***Detection of Schistosoma DNA*** using ITS-2 Real-time PCR processed at Zambart laboratories in Lusaka, Zambia [31 38]. From the DNA already extracted for PCR additional well-validated qPCR assays will be used [39] to characterize the vaginal microbiota by quantifying key species and 2) ***Detection of Schistosoma DNA using RPA***. Up to 2 mL of fluid from vaginal lavage is stored at -20°C and shipped to Lusaka. This assay is processed at Zambart laboratory in Zambia [40 41] and 3) ***Inflammatory Markers***. Up to 3 mL of fluid from vaginal lavage is stored at -80 ^{o}C and shipped to Lusaka. [25 31 42].

<< Insert Table 1 here>>

Development of a multiplex assay for *S. haematobium* and HR-HPV testing

In a exploratory study we will aim to integrate into one molecular assay the RPA for *Sh* and HPV to increase accessibility and feasibility of molecular dual pathogen screenings after encouraging results on the validaty of the CVL RPA assay against standard real-time PCR[43], which could be coupled with oncogenic HPV genotypes. [44].

Qualitative assessment

In-Depth feasibility and acceptability of integrated sampling, testing and treatment

For peer review only - http://bmjopen.bmj.com/site/about/guidelines.xhtml

For peer review only

Previous work has documented the acceptability of performing multiple genital swabs at a single encounter [27]. For the current proposal, in-depth cognitive interviews will be delivered to a subset of participants to evaluate the acceptability of the multi-pathogen self-swabbing strategy (three genital swabs and one oral swab) [45].

STATISTICAL ANALYSIS

To assess the performance of genital self-sampling compared to healthcare provider sampling for the detection of *Sh,* sensitivity, specificity, PPV and NPV will be calculated together with 95% confidence intervals. Results will be presented overall and stratified by geographic setting. To explore associations of baseline FGS with prevalence of cervical precancer at baseline and cervical precancer incidence at 12 and 24 months, logistic regression will be used to estimate odds ratios. Associations between baseline FGS and HR-HPV persistence at 12 months will be estimated with generalized estimating equation to account for multiple HR-HPV infection and multiple infection states (persistence and clearance)[46]. The association of FGS with HIV acquisition will be assessed using Poisson regression, controlling for key confounders.

Other outcome measures obtained in the study will include 1)HIV incidence rate, HR-HPV clearance and persistence, prevalence and incidence of cervical precancer (histology confirmed CIN2 or CIN3+), measures of association between different exposures (reproductive tract infections). 2). Acceptability of multiple genital self-sampling interventions 3). Rates of ART initiation, continuation of treatment and access to care 4). Cervical precancer diagnosis and treatment, testing and management of STIs. 4). Presence or absence of cervicovaginal microbiota in women with FGS, HPV and STIs. Expressed as relative abundance (percentage abundance of each species), genomic concentration per microorganism (genome equivalents per mL). 5) Sensitivity, specificity, PPV, NPV of visual inspection of the cervix according to FGS status and Integrated molecular field-applicable testing platform for *Sh* and HR-HPV

Quality control

Several procedures have been employed to ensure the quality of the study data, maximizing the validity and reliability of the data obtained and of the evaluation of the results: These include the following (a) 10

For peer review only - http://bmjopen.bmj.com/site/about/guidelines.xhtml

% of all samples will be reviewed blindly by experts at different institutions; parasitology (Liverpool School of Tropical Medicine, Liverpool, UK), PCR (Natural History Museum, London, UK), colposcopic images (University of Zambia, Lusaka, Zambia)

Economic analysis

A full economic costing from a provider's and patient's perspective is ongoing alongside data collection. Cost data is collected using a mixed method of bottom-up and top-down as following established costing guidelines [47]. This allows us to estimate the total intervention cost, and unit costs per: woman screened, FGS infection detected, per case of CIN2+ detected, per person linked to care, per person successfully treated overall and per pathogen. From this we can model the costs across a set of scale-up scenarios to understand the budget implications of introducing widescale self-screening. Using decision health economics models and the study outcomes we can use cost estimates to understand under which circumstances joint self-sampling for FGS, HPV, STIs and HIV is cost-effective. This will be relevant for informing the maximum threshold diagnostic kit price based on its potential cost-effectiveness [48]. This will also inform MoH and policy makers on the most cost-effective screening strategy to implement for FGS screening. This will be the first economic evaluation for FGS.

DISCUSSION

Sexual and reproductive health is a critical area of development highlighted in the United Nations Sustainable Development Goal 3. There is an estimated 40 million women unknowingly living with FGS, with dire consequences for their sexual and reproductive lives that include infertility, pain with sexual relations, ectopic pregnancies and increased risk of HIV acquisition and cervical precancer development, mostly in countries with high prevalence of STIs. This study will provide high quality evidence on the validity, acceptability, uptake and costs of integrating community based multiple-pathogen *genital self-sampling* (*Sh*, HR-HPV, STIs) and *oral self-test* (HIV) into *one-stop intervention*. Findings will help inform policy and practice at local and regional levels. Importantly, this study brings together existing and new partnerships for a multidisciplinary investigative approach, to explore the value of integration of reproductive health screening practices through self-sampling and testing. The baseline results will

For peer review only - http://bmjopen.bmj.com/site/about/guidelines.xhtml

provide further validation of self-swabs as an acceptable sampling strategy for FGS diagnosis in a larger sample population and with a wider scope than previously performed. Its longitudinal approach will also provide an allied impact assessment on the incidence of cervical precancer, HIV and STIs and early HIV detection and access to treatment for the wider improvement of women's SRH in Zambia. There is growing evidence of a possible link between FGS and cervical cancer [15]. This clear interplay between genital infections highlights the urgent need to bolster programmes that jointly integrate strategies to screen and detect multiple genital infections with simple, affordable and as close to the point-of-care as possible.

Genital self-sampling and self-testing through community based programmes are widely accepted strategies for the detection of devastating diseases like HIV in both adults and adolescents [8 31 49]. HIV self-testing programmes have been shown to be cost-effective. Genital self-sampling has enhanced access to health services among hard-to-reach populations such as young people [31], and those who do not regularly access health screening services [1 50]. It also empowers women and protect their privacy since self-swabs can be performed at home or in other private places and increases compliance with testing. A high proportion of women, including those from resource-limited settings have been found to prefer vaginal specimen self-collection [51] compared with clinic-based sampling. In addition to acceptability, two other factors make genital self-sampling advantageous; 1) the availability of vaginal self-sampling is effective for improving participation in specific SRH screening programmes and 2) the sensitivity of PCR-based assays on self-collected specimens compares favourably with physician-performed sampling [1].

The synergistic approach proposed in this study is justified by the known and suspected interactions of the different genital co-infections. FGS is associated with genital inflammation, friable blood vessels and contact bleeding [52-54]. The disruptions of the mucosal surface in FGS may place women at risk for HIV, as has been found for several STIs [55-57]. Women with urogenital schistosomiasis had a 3-4-fold higher odds of prevalent HIV in cross-sectional studies in Zimbabwe, Tanzania and Mozambique [58-62]. However, FGS as a risk factor for HIV is not yet integrated in ongoing screening programmes, including HIV self-testing strategies [63-65].

For peer review only

For peer review only - http://bmjopen.bmj.com/site/about/guidelines.xhtml

Current FGS diagnostic strategies are limited because they require expertise and equipment that may not be readily available in low income settings. Several diagnostics will be tested in this study to provide 'real-world' data on the scalability of the different approaches. Hand-held colposcopy as a low-cost device has been used in both cervical cancer screening and FGS studies [4 66]. However, expert review is still required for the identification of characteristic FGS lesions and inter-rater correlation is poor [67]. In addition, in this study, testing for the DNA detection of different pathogens will be performed with conventional molecular assays that are field deployable [32 40]. Following on the positive correlation between RPA assay from CVL against standard real-time PCR, [68] in the Schista study we will aim to develop a multiplex RPA assay for *Sh* and HR-HPV to increase accessibility and feasibility of dual pathogen molecular screenings.

To make interventions ultimately sustainable these need to be cost-effective. In this study we aim to assess for the first time the observed costs of integrating community based multiple-pathogen genital self-sampling (*Sh*, HPV, STIs) and oral self-test (HIV) in a *one-stop* intervention and project costs at scale for the justification of the diagnostic kit price based on its impact on disease prevalence [26].

To conclude, the Schista study has a number of potential benefits at both individual and community levels including diagnosis and treatment of previously undiagnosed *S. haematobium* infection as well as diagnosis, treatment and linkage to care for cervical cancer, HIV and STIs. Multi-pathogen self-sampling and testing constitutes an unmissable opportunity to offer potentially life-changing health services closer to the users [6].

For peer review only

For peer review only - http://bmjopen.bmj.com/site/about/guidelines.xhtml

DECLARATIONS

Participant involvement

Participants who are being recruited to this study did not take part in the design or implementation of the study. They are however deeply involved finding solutions for better follow up through focus groups discussions and mobilisation activities in the community.

Competing interest statement

The authors declare they have no competing interests.

Funding statement

This study is funded through a UKRI Future Leaders Fellowship (MR/T041900/1) awarded to Prof. AL Bustinduy. Prof E Webb and Prof R Hayes received funding from MRC Grant Reference MR/K012126/1. This award is jointly funded by the UK Medical Research Council (MRC) and the UK Department for International Development (DFID) under the MRC/DFID Concordat agreement and is also part of the EDCTP2 programmme supported by the European Union. Funders had no role in study design, data collection and analysis, decision to publish, or preparation of the manuscript.

Authors' Contributions

AB originally conceived and designed the study in consultation with PM, HA, BW, IH, FTP and RH. KS wrote the first draft of this manuscript. BW, HK and AS advised on primary and some secondary objectives, FTP and OL conceived the economic analysis, EW and NK helped design the statistical framework of the study and contributed to all drafts of the manuscript. BW, LVL, JF, GVD and PC contributed to the laboratory component of the study. RN and VB contributed to the design of secondary objectives. All authors read and approved the final manuscript.

Acknowledgements

We would like to thank and acknowledge all the participants, Schista! study fieldworkers and midwives. and the wider Schista! study team who contributed towards the design of the study. Also, our partners in Zambia including the Ministry of Health and District Health Management Teams; the administrative and support teams at Zambart and LSHTM.

For peer review only

For peer review only - http://bmjopen.bmj.com/site/about/guidelines.xhtml

REFERENCES

1. Kjetland EF, Kurewa EN, Mduluza T, et al. The first community-based report on the effect of genital Schistosoma haematobium infection on female fertility. *Fertil Steril* 2010;94(4):1551-3. doi: 10.1016/j.fertnstert.2009.12.050
2. Bustinduy AL, Randriansolo B, Sturt AS, et al. Chapter One - An update on female and male genital schistosomiasis and a call to integrate efforts to escalate diagnosis, treatment and awareness in endemic and non-endemic settings: The time is now. In: Rollinson D, Stothard R, eds. Advances in Parasitology: Academic Press 2022:1-44.
3. Hotez PJ, Harrison W, Fenwick A, et al. Female genital schistosomiasis and HIV/AIDS: Reversing the neglect of girls and women. *PLoS Negl Trop Dis* 2019;13(4):e0007025. doi: 10.1371/journal.pntd.0007025 [published Online First: 2019/04/05]
4. Sturt AS, Webb EL, Phiri CR, et al. Genital self-sampling compared with cervicovaginal lavage for the diagnosis of female genital schistosomiasis in Zambian women: The BILHIV study. *PLoS Negl Trop Dis* 2020;14(7):e0008337. doi: 10.1371/journal.pntd.0008337 [published Online First: 2020/07/15]
5. Mbatha JN, Galappaththi-Arachchige HN, Mtshali A, et al. Self-sampling for human papillomavirus testing among rural young women of KwaZulu-Natal, South Africa. *BMC Res Notes* 2017;10(1):702. doi: 10.1186/s13104-017-3045-3 [published Online First: 2017/12/07]
6. Francis SC, Mthiyane TN, Baisley K, et al. Prevalence of sexually transmitted infections among young people in South Africa: A nested survey in a health and demographic surveillance site. *PLoS Med* 2018;15(2):e1002512. doi: 10.1371/journal.pmed.1002512 [published Online First: 2018/02/28]
7. Wall KM, Kilembe W, Vwalika B, et al. Schistosomiasis is associated with incident HIV transmission and death in Zambia. *PLoS Negl Trop Dis* 2018;12(12):e0006902. doi: 10.1371/journal.pntd.0006902 [published Online First: 2018/12/14]
8. Kjetland EF, Ndhlovu PD, Gomo E, et al. Association between genital schistosomiasis and HIV in rural Zimbabwean women. *AIDS* 2006;20(4):593-600. doi: 10.1097/01.aids.0000210614.45212.0a
9. Downs JA, Dupnik KM, van Dam GJ, et al. Effects of schistosomiasis on susceptibility to HIV-1 infection and HIV-1 viral load at HIV-1 seroconversion: A nested case-control study. *PLoS Negl Trop Dis* 2017;11(9):e0005968. doi: 10.1371/journal.pntd.0005968 [published Online First: 2017/09/26]
10. Downs JA, Mguta C, Kaatano GM, et al. Urogenital schistosomiasis in women of reproductive age in Tanzania's Lake Victoria region. *Am J Trop Med Hyg* 2011;84(3):364-9. doi: 10.4269/ajtmh.2011.10-0585
11. Wall KM, Kilembe W, Vwalika B, et al. Schistosomiasis is associated with incident HIV transmission and death in Zambia. *PLOS Neglected Tropical Diseases* 2018;12(12):e0006902. doi: 10.1371/journal.pntd.0006902
12. Kjetland EF, Ndhlovu PD, Mduluza T, et al. The effects of genital schistosoma haematobium on human papillomavirus and the development of cervical neoplasia after five years in a Zimbabwean population. *Eur J Gynaecol Oncol* 2010;31(2):169-73.
13. Rafferty H, Sturt AS, Phiri CR, et al. Association between cervical dysplasia and female genital schistosomiasis diagnosed by genital PCR in Zambian women. *BMC Infect Dis* 2021;21(1):691. doi: 10.1186/s12879-021-06380-5 [published Online First: 2021/07/19]
14. Vlassoff C, Arogundade K, Patel K, et al. Improving the Response of Health Systems to Female Genital Schistosomiasis in Endemic Countries through a Gender-Sensitive Human Rights-Based Framework. *Diseases* 2022;10(4):125.
15. Engels D, Hotez PJ, Ducker C, et al. Integration of prevention and control measures for female genital schistosomiasis, HIV and cervical cancer. *Bull World Health Organ* 2020;98(9):615-24. doi: 10.2471/blt.20.252270 [published Online First: 2020/10/06]

For peer review only

16. Bray F, Ferlay J, Soerjomataram I, et al. Global cancer statistics 2018: GLOBOCAN estimates of incidence and mortality worldwide for 36 cancers in 185 countries. *CA Cancer J Clin* 2018;68(6):394-424. doi: 10.3322/caac.21492 [published Online First: 2018/09/13]
17. Bruni L AG, Serrano B, Mena M, Gómez D, Muñoz J, Bosch FX, de Sanjosé S. . Human Papillomavirus and Related Diseases in Zambia. Summary Report 17 June 2019.: ICO/IARC Information Centre on HPV and Cancer (HPV Information Centre). 2019 [accessed 15th October 2019.
18. Bruni L, Serrano B, Roura E, et al. Cervical cancer screening programmes and age-specific coverage estimates for 202 countries and territories worldwide: a review and synthetic analysis. *Lancet Glob Health* 2022;10(8):e1115-e27. doi: 10.1016/s2214-109x(22)00241-8 [published Online First: 2022/07/16]
19. Bruni L, Saura-Lázaro A, Montoliu A, et al. HPV vaccination introduction worldwide and WHO and UNICEF estimates of national HPV immunization coverage 2010-2019. *Prev Med* 2021;144:106399. doi: 10.1016/j.ypmed.2020.106399 [published Online First: 2021/01/04]
20. World Health Organization (2021). WHO guideline for screening and treatment of cervical pre-cancer lesions for cervical cancer prevention, 2nd ed. World Health Organization. https://apps.who.int/iris/handle/10665/342365. License: CC BY-NC-SA 3.0 IGO.
21. Arbyn M, Sankaranarayanan R, Muwonge R, et al. Pooled analysis of the accuracy of five cervical cancer screening tests assessed in eleven studies in Africa and India. *Int J Cancer* 2008;123(1):153-60. doi: 10.1002/ijc.23489 [published Online First: 2008/04/12]
22. Arbyn M, Simon M, Peeters E, et al. 2020 list of human papillomavirus assays suitable for primary cervical cancer screening. *Clin Microbiol Infect* 2021;27(8):1083-95. doi: 10.1016/j.cmi.2021.04.031 [published Online First: 2021/05/12]
23. Arbyn M, Smith SB, Temin S, et al. Detecting cervical precancer and reaching underscreened women by using HPV testing on self samples: updated meta-analyses. *BMJ* 2018;363:k4823. doi: 10.1136/bmj.k4823 [published Online First: 2018/12/07]
24. Mezei AK, Pedersen HN, Sy S, et al. Community-based HPV self-collection versus visual inspection with acetic acid in Uganda: a cost-effectiveness analysis of the ASPIRE trial. *BMJ Open* 2018;8(6):e020484. doi: 10.1136/bmjopen-2017-020484 [published Online First: 2018/06/14]
25. Lunny C, Taylor D, Hoang L, et al. Self-Collected versus Clinician-Collected Sampling for Chlamydia and Gonorrhea Screening: A Systemic Review and Meta-Analysis. *PLoS One* 2015;10(7):e0132776. doi: 10.1371/journal.pone.0132776
26. Mangenah C, Mwenge L, Sande L, et al. Economic cost analysis of door-to-door community-based distribution of HIV self-test kits in Malawi, Zambia and Zimbabwe. *J Int AIDS Soc* 2019;22 Suppl 1:e25255. doi: 10.1002/jia2.25255 [published Online First: 2019/03/26]
27. Rutty Phiri C, Sturt AS, Webb EL, et al. Acceptability and feasibility of genital self-sampling for the diagnosis of female genital schistosomiasis: a cross-sectional study in Zambia. *Wellcome Open Res* 2020;5:61. doi: 10.12688/wellcomeopenres.15482.2 [published Online First: 2020/11/10]
28. Zachary D, Mwenge L, Muyoyeta M, et al. Field comparison of OraQuick ADVANCE Rapid HIV-1/2 antibody test and two blood-based rapid HIV antibody tests in Zambia. *BMC Infect Dis* 2012;12:183. doi: 10.1186/1471-2334-12-183 [published Online First: 2012/08/09]
29. Campbell L, Woods V, Lloyd T, et al. Evaluation of the OSOM Trichomonas rapid test versus wet preparation examination for detection of Trichomonas vaginalis vaginitis in specimens from women with a low prevalence of infection. *J Clin Microbiol* 2008;46(10):3467-9. doi: 10.1128/JCM.00671-08 [published Online First: 2008/08/08]
30. van Dam GJ, de Dood CJ, Lewis M, et al. A robust dry reagent lateral flow assay for diagnosis of active schistosomiasis by detection of Schistosoma circulating anodic antigen. *Exp Parasitol* 2013;135(2):274-82. doi: 10.1016/j.exppara.2013.06.017 [published Online First: 2013/07/16]
31. Kjetland EF, Hove RJ, Gomo E, et al. Schistosomiasis PCR in vaginal lavage as an indicator of genital Schistosoma haematobium infection in rural Zimbabwean women. *Am J Trop Med Hyg* 2009;81(6):1050-5. doi: 10.4269/ajtmh.2009.09-0081
32. Garrett N, Mitchev N, Osman F, et al. Diagnostic accuracy of the Xpert CT/NG and OSOM Trichomonas Rapid assays for point-of-care STI testing among young women in South Africa: a

For peer review only

For peer review only - http://bmjopen.bmj.com/site/about/guidelines.xhtml

cross-sectional study. *BMJ Open* 2019;9(2):e026888. doi: 10.1136/bmjopen-2018-026888 [published Online First: 2019/02/21]

33. Ministry of Health. National cancer control strategic plan 2016-2021. Lusaka: Zambia 2017 [Available from: https://www.iccp-portal.org/system/files/plans/NCCSP%20Final%20Version%20Zambia.pdf, accessed 5th October 2022.
34. Parham GP, Mwanahamuntu MH, Kapambwe S, et al. Population-level scale-up of cervical cancer prevention services in a low-resource setting: development, implementation, and evaluation of the cervical cancer prevention program in Zambia. *PLoS One* 2015;10(4):e0122169. doi: 10.1371/journal.pone.0122169 [published Online First: 2015/04/18]
35. Woodman CB, Collins S, Winter H, et al. Natural history of cervical human papillomavirus infection in young women: a longitudinal cohort study. *Lancet* 2001;357(9271):1831-6. doi: 10.1016/S0140-6736(00)04956-4 [published Online First: 2001/06/19]
36. Mumba JM, Kasonka L, Owiti OB, et al. Cervical cancer diagnosis and treatment delays in the developing world: Evidence from a hospital-based study in Zambia. *Gynecol Oncol Rep* 2021;37:100784. doi: 10.1016/j.gore.2021.100784 [published Online First: 2021/06/08]
37. Corstjens PL, van Lieshout L, Zuiderwijk M, et al. Up-converting phosphor technology-based lateral flow assay for detection of Schistosoma circulating anodic antigen in serum. *J Clin Microbiol* 2008;46(1):171-6. doi: 10.1128/JCM.00877-07
38. Obeng BB, Aryeetey YA, de Dood CJ, et al. Application of a circulating-cathodic-antigen (CCA) strip test and real-time PCR, in comparison with microscopy, for the detection of Schistosoma haematobium in urine samples from Ghana. *Ann Trop Med Parasitol* 2008;102(7):625-33. doi: 10.1179/136485908X337490
39. Jespers V, van de Wijgert J, Cools P, et al. The significance of Lactobacillus crispatus and L. vaginalis for vaginal health and the negative effect of recent sex: a cross-sectional descriptive study across groups of African women. *BMC Infect Dis* 2015;15:115. doi: 10.1186/s12879-015-0825-z [published Online First: 2015/04/17]
40. Rosser A, Rollinson D, Forrest M, et al. Isothermal Recombinase Polymerase amplification (RPA) of Schistosoma haematobium DNA and oligochromatographic lateral flow detection. *Parasit Vectors* 2015;8:446. doi: 10.1186/s13071-015-1055-3
41. Utzinger J, Becker SL, van Lieshout L, et al. New diagnostic tools in schistosomiasis. *Clin Microbiol Infect* 2015;21(6):529-42. doi: 10.1016/j.cmi.2015.03.014
42. Sturt AS, Webb EL, Patterson C, et al. Cervicovaginal Immune Activation in Zambian Women With Female Genital Schistosomiasis. *Front Immunol* 2021;12:620657. doi: 10.3389/fimmu.2021.620657 [published Online First: 2021/03/20]
43. Archer J, Patwary FK, Sturt AS, et al. Validation of the isothermal Schistosoma haematobium Recombinase Polymerase Amplification (RPA) assay, coupled with simplified sample preparation, for diagnosing female genital schistosomiasis using cervicovaginal lavage and vaginal self-swab samples. *PLOS Neglected Tropical Diseases* 2022;16(3):e0010276. doi: 10.1371/journal.pntd.0010276
44. Ma B, Fang J, Wang Y, et al. Isothermal Method of a Recombinase Polymerase Amplification Assay for the Detection of Most Common High-Risk Human Papillomavirus Type 16 and Type 18 DNA. *Clin Lab* 2017;63(1):27-38. doi: 10.7754/Clin.Lab.2016.160325 [published Online First: 2017/02/07]
45. Simwinga M KM, Dacombe RJ, Kayira L, Muzumara A, Johnson CC, et al. Ability to understand and correctly follow HIV self-test kit instructions for use: applying the cognitive interview technique in Malawi and Zambia. *J Int AIDS Soc*;22 Suppl 1:e25253
46. Xue X, Gange SJ, Zhong Y, et al. Marginal and Mixed-Effects Models in the Analysis of Human Papillomavirus Natural History Data. *Cancer Epidemiology, Biomarkers & Prevention* 2010;19:159 - 69.
47. Vassall A, Sweeney, S., Kahn, J., Gomez, G. B., Bollinger, L., Marseille, E., … & Levin, C, . Reference Case for Estimating the Costs of Global Health Services and Interventions. Global

For peer review only - http://bmjopen.bmj.com/site/about/guidelines.xhtml

Health Cost Consortium. 2017 [Available from: https://ghcosting.org/pages/standards/reference_case accessed 15th March 2022.
48. Terris-Prestholt F, Foss AM, Cox AP, et al. Cost-effectiveness of tenofovir gel in urban South Africa: model projections of HIV impact and threshold product prices. *BMC Infect Dis* 2014;14:14. doi: 10.1186/1471-2334-14-14 [published Online First: 2014/01/11]
49. Kjetland EF, Poggensee G, Helling-Giese G, et al. Female genital schistosomiasis due to Schistosoma haematobium. Clinical and parasitological findings in women in rural Malawi. *Acta Trop* 1996;62(4):239-55.
50. Pillay P, Downs JA, Changalucha JM, et al. Detection of Schistosoma DNA in genital specimens and urine: A comparison between five female African study populations originating from S. haematobium and/or S. mansoni endemic areas. *Acta Tropica* 2020;204:105363. doi: https://doi.org/10.1016/j.actatropica.2020.105363
51. Helling-Giese G, Kjetland EF, Gundersen SG, et al. Schistosomiasis in women: manifestations in the upper reproductive tract. *Acta Tropica* 1996;62(4):225-38. doi: https://doi.org/10.1016/S0001-706X(96)00025-3
52. Yeh HY, Mitchell PD. Ancient Human Parasites in Ethnic Chinese Populations. *The Korean journal of parasitology* 2016;54(5):565-72. doi: 10.3347/kjp.2016.54.5.565 [published Online First: 2016/11/18]
53. Barakat RM. Epidemiology of Schistosomiasis in Egypt: Travel through Time: Review. *Journal of advanced research* 2013;4(5):425-32. doi: 10.1016/j.jare.2012.07.003 [published Online First: 2013/09/01]
54. Madden F. A case of Bilharzia of the vagina. *Lancet* 1899;1:1716-10.
55. Kaul R, Pettengell C, Sheth PM, et al. The genital tract immune milieu: an important determinant of HIV susceptibility and secondary transmission. *J Reprod Immunol* 2008;77(1):32-40.
56. Masson L, Passmore JA, Liebenberg LJ, et al. Genital inflammation and the risk of HIV acquisition in women. *Clin Infect Dis* 2015;61(2):260-9. doi: 10.1093/cid/civ298 [published Online First: 2015/04/23]
57. Kjetland EF, Hegertun IE, Baay MF, et al. Genital schistosomiasis and its unacknowledged role on HIV transmission in the STD intervention studies. *International journal of STD & AIDS* 2014;25(10):705-15. doi: 10.1177/0956462414523743 [published Online First: 2014/03/14]
58. Kjetland EF, Leutscher PD, Ndhlovu PD. A review of female genital schistosomiasis. *Trends Parasitol* 2012;28(2):58-65. doi: 10.1016/j.pt.2011.10.008 [published Online First: 2012/01/17]
59. Downs JA, Mguta C, Kaatano GM, et al. Urogenital schistosomiasis in women of reproductive age in Tanzania's Lake Victoria region. *Am J Trop Med Hyg* 2011;84(3):364-9. doi: 10.4269/ajtmh.2011.10-0585 [published Online First: 2011/03/03]
60. Hotez PJ, Fenwick A, Kjetland EF. Africa's 32 cents solution for HIV/AIDS. *PLoS Negl Trop Dis* 2009;3(5):e430. doi: 10.1371/journal.pntd.0000430
61. Brodish PH, Singh K. Association between *Schistosoma haematobium* exposure and Human Immunodeficiency Virus infection among females in Mozambique. *Am J Trop Med Hyg* 2016;94(5):1040-4. doi: 10.4269/ajtmh.15-0652 [published Online First: 2016/03/16]
62. Ndeffo Mbah ML, Poolman EM, Drain PK, et al. HIV and Schistosoma haematobium prevalences correlate in sub-Saharan Africa. *Tropical Medicine and International Health* 2013 doi: 10.1111/tmi.12165
63. Berge ST, Kabatereine NB, Gundersen SG, et al. Generic praziquantel in South Africa: The necessity for policy change to avail cheap, safe and efficacious schistosomiasis drugs to the poor, rural population *Southern Afr J Epidemiol Infect* 2011;26(1):22-25.
64. Lothe A. Treating Bilharzia among High School Pupils. A study of opportunities and constraints for treating Bilharzia among high school pupils in Ugu district, South Africa. Agder University College, 2012.
65. Ndhlovu PD, Mduluza T, Kjetland EF, et al. Prevalence of urinary schistosomiasis and HIV in females living in a rural community of Zimbabwe: does age matter? *Transactions of the Royal Society of Tropical Medicine and Hygiene* 2007;101(5):433-38. doi: 10.1016/j.trstmh.2006.08.008 [published Online First: 2006/10/27]

For peer review only

66. Taghavi K, Moono M, Mwanahamuntu M, et al. Screening test accuracy to improve detection of precancerous lesions of the cervix in women living with HIV: a study protocol. *BMJ Open* 2020;10(12):e037955. doi: 10.1136/bmjopen-2020-037955 [published Online First: 20201218]
67. Sturt A, Bristowe H, Webb E, et al. Visual diagnosis of female genital schistosomiasis in Zambian women from hand-held colposcopy: agreement of expert image review. *Wellcome Open Res* 2023;8:14. doi: 10.12688/wellcomeopenres.18737.1 [published Online First: 20230110]
68. Archer J, Patwary FK, Sturt AS, et al. Validation of the isothermal Schistosoma haematobium Recombinase Polymerase Amplification (RPA) assay, coupled with simplified sample preparation, for diagnosing female genital schistosomiasis using cervicovaginal lavage and vaginal self-swab samples. *PLoS Negl Trop Dis* 2022;16(3):e0010276. doi: 10.1371/journal.pntd.0010276 [published Online First: 2022/03/15]

For peer review only

Supplementary Information

Supplemental figure:3D models for the teaching of self sampling

For peer review only - http://bmjopen.bmj.com/site/about/guidelines.xhtml

Figure 1: A schematic study flow

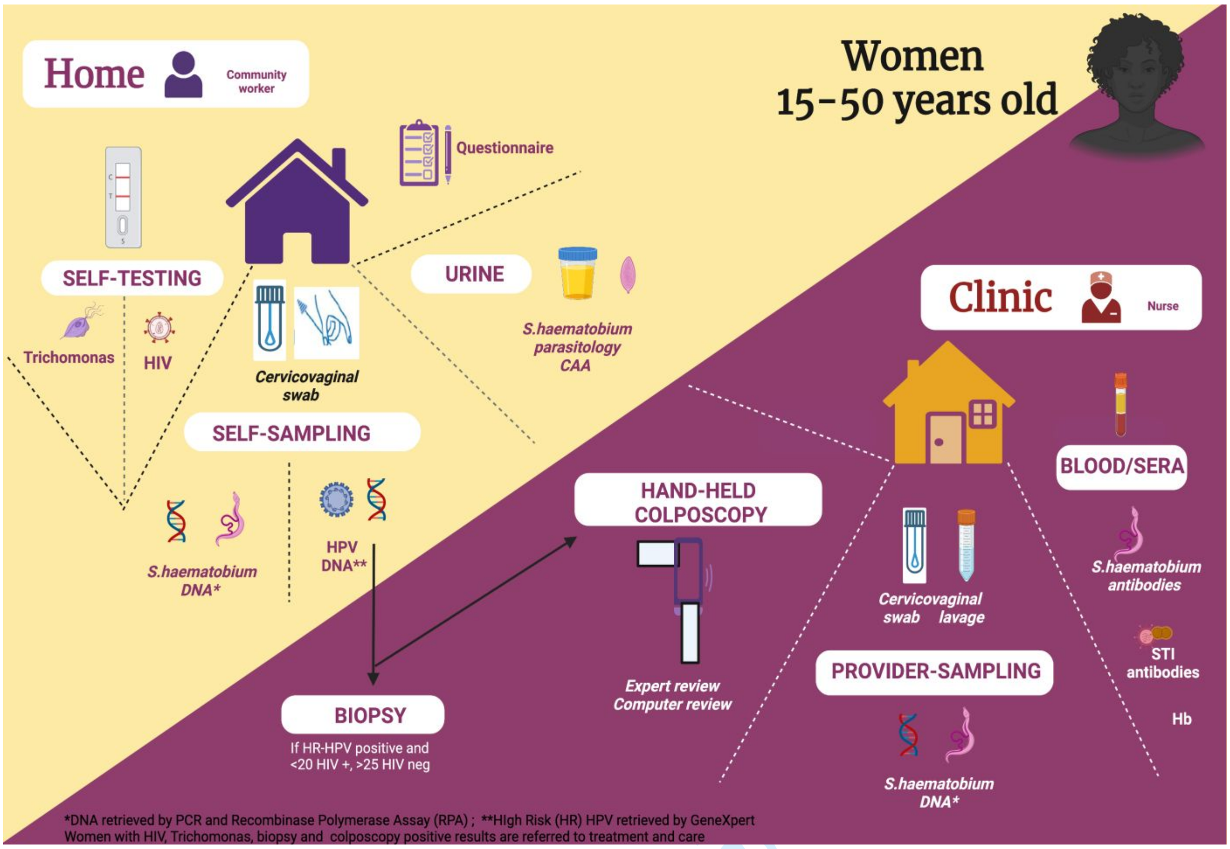

For peer review only - http://bmjopen.bmj.com/site/about/guidelines.xhtml

Figure 2 . Flowchart for cervical precancer determination in HIV negative and women living with HIV

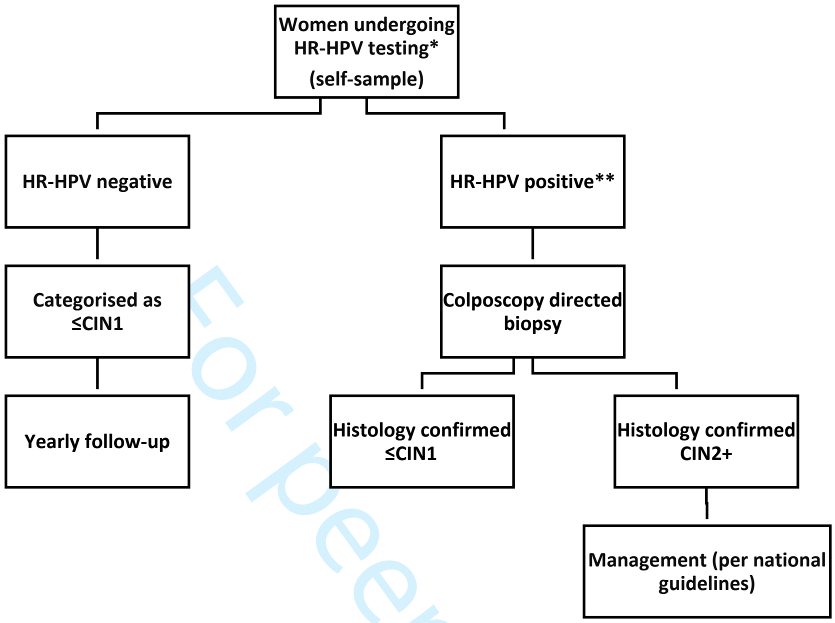

**HR-HPV detected using Xpert; **HR-HPV positive WLHIV aged 20 years and older and HIV-negative women aged 25 years and older only.*

For peer review only

Table 1: Sample storage and processing for the Schista study.

Sample	Storage	Sample Processing	Location of processing
**Self-collected swab**<br>**Provider-collected swab**	Room temperature (Stored in Primestore media®)	Sh DNA (PCR, RPA)<br>GeneXpert- High Risk HPV DNA*	Zambart (Zambia)
**Urine for microscopy**	Room temperature	Sh eggs<br>Urine haemastix	District Laboratory (Kafue, Livingstone, Zambia)
**Urine stored for CAA**	4 °C	Circulating Anodic Antigen	Leiden University Medical Centre (The Netherlands)
**Cervicovaginal lavage**	-20 °C	Sh DNA (PCR, RPA)<br>GeneXpert- CT/NG<br>Microbiota	Zambart (Zambia)
**Blood for serology**	-20 °C	Sh, STI Antibody	Zambart (Zambia)
**Colposcopy images**	Encrypted server	Expert Review<br>Computer Analysis	Zambia<br>London School of Hygien & Tropical Medicine (UK)

*High risk HPV by self-collected swab only

For peer review only

For peer review only - http://bmjopen.bmj.com/site/about/guidelines.xhtml

List of Abbreviations

AE	Adverse Event
EDC	Electronic Data Capture
FGS	Female Genital Schistosomiasis
CAA	Circulating anodic antigen
CIN	Cervical intraepithelial neoplasia
ICC	Invasive Cervical Cancer
LLETZ	Large loop excision of the transformation zone
CW	Community workers
HR-HPV	High Risk Human Papilloma Virus
ODK	Open Data Kit
PCR	Polymerase Chain Reaction
RPA	Recombinase Polymerase Amplification
SCW	Schista Community Worker
SRH	Sexual and Reproductive Health
Sh	*Schistosoma haematobium*
SSA	Sub-Saharan Africa
STI	Sexually transmitted infections
Tv	*Trichomonas vaginalis*
VIA	Visual inspection with acetic acid

For peer review only

Supplementary figure 1: 3 D models for the teaching of self-sampling

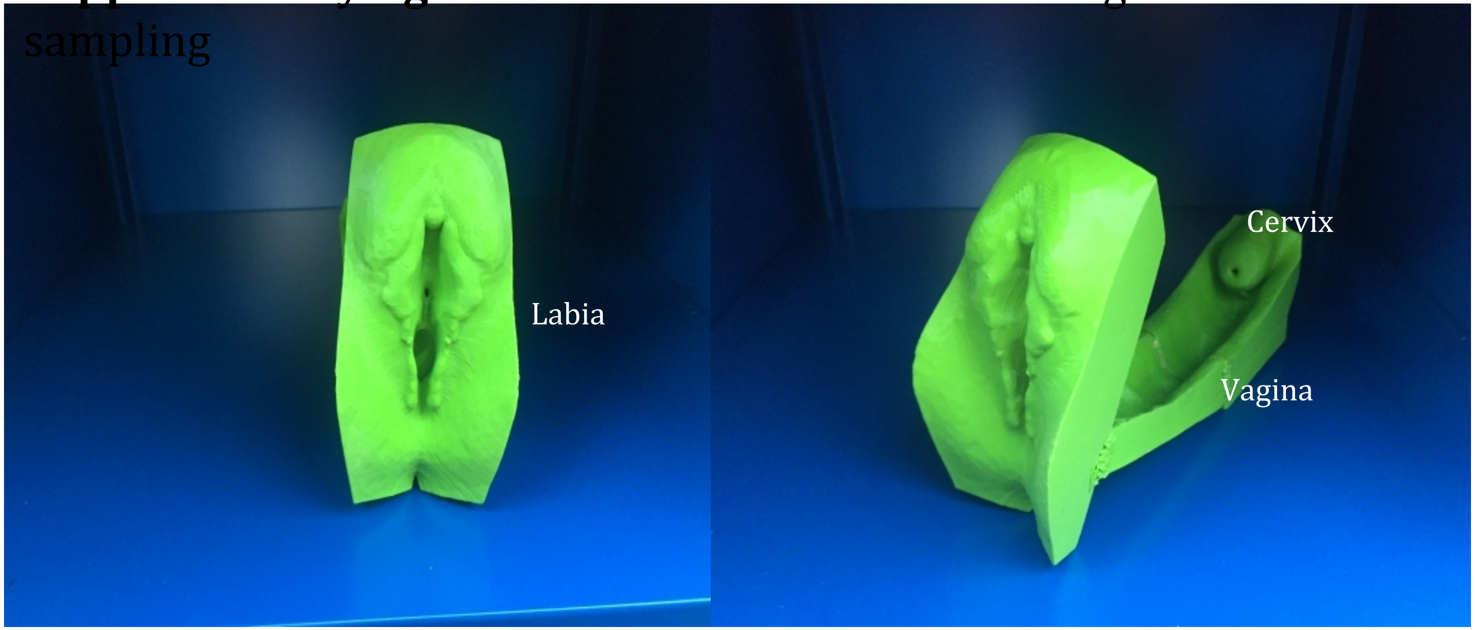

BMJ Open

The Zipime-Weka-Schista study protocol: a longitudinal cohort study and economic evaluation of an integrated home-based approach for genital multi-pathogen screening in women, including female genital schistosomiasis, HPV, Trichomonas and HIV in Zambia

Journal:	*BMJ Open*
Manuscript ID	bmjopen-2023-080395.R1
Article Type:	Protocol
Date Submitted by the Author:	20-Mar-2024

Complete List of Authors:	Shanaube, Kwame; Zambart<br>Ndubani, Rhoda; Zambart<br>Kelly, Helen; London School of Hygiene and Tropical Medicine, Clinical Research Department<br>Webb, Emily; London School of Hygiene and Tropical Medicine, Faculty of Infectious and Tropical Diseases<br>Mayaud, Philippe; London School of Hygiene & Tropical Medicine Faculty of Infectious and Tropical Diseases, Clinical Research<br>Lamberti, Olimpia; London School of Hygiene and Tropical Medicine, Clinical Research Department<br>Fitzpatrick, Jennifer; Zambart<br>Kasese, Nkatya; Zambart<br>Sturt, Amy; Veterans Affairs Health Care System, Department of Infectious Diseases<br>Van Lieshout, Lisette; Leiden University Medical Center<br>Van Dam, Govert; Leiden University Medical Center<br>Corstjens, Paul; Leiden University Medical Center, Department of Cell and Chemical Biology<br>Kosloff, Barry; Zambart<br>Bond, Virginia; London School of Hygiene & Tropical Medicine Centre of Global Change and Health<br>Hayes, Richard; London School of Hygiene and Tropical Medicine, Epidemiology and Population Health<br>Terris-Prestholt, Fern; London School of Hygiene & Tropical Medicine<br>Webster, Bonnie; Natural History Museum<br>Vwalika, Bellington; University of Zambia School of Medicine, Department of Obstetrics & Gynaecology<br>Hansingo, Isaiah; Livingstone Central Hospital, Gynecology<br>Ayles, Helen; London School of Hygiene & Tropical Medicine; Zambart<br>Bustinduy, Amaya; London School of Hygiene and Tropical Medicine, Clinical Research; London School of Hygiene & Tropical Medicine

**Primary Subject Heading**:	Global health
Secondary Subject Heading:	Sexual health

For peer review only - http://bmjopen.bmj.com/site/about/guidelines.xhtml

Keywords:	Adolescent, PUBLIC HEALTH, SEXUAL MEDICINE, Molecular diagnostics < INFECTIOUS DISEASES, PARASITOLOGY, Colposcopy < GYNAECOLOGY

SCHOLARONE™
Manuscripts

The Zipime-Weka-Schista study protocol: a longitudinal cohort study and economic evaluation of an integrated home-based approach for genital multi-pathogen screening in women, including female genital schistosomiasis, HPV, Trichomonas and HIV in Zambia

*Kwame Shanaube[1], Rhoda Ndubani[1], Helen Kelly[2], Emily L. Webb[3], Philippe Mayaud[2], Olimpia Lamberti[2], Jennifer Fitzpatrick[1], Nkatya Kasese[1], Amy Sturt[5], Lisette van Lieshout[6], Govert J. van Dam[6], Paul L.A.M. Corstjens[7], Barry Kosloff[18], Virginia Bond[1,2], Richard Hayes[3], Fern Terris-Prestholt[9], Bonnie Webster[10], Bellington Vwalika[11], Isaiah Hansingo[4] , Helen Ayles[1,2], Amaya L. Bustinduy[2]

1. Zambart, Lusaka, Zambia
2. Department of Clinical Research, London School of Hygiene & Tropical Medicine, Keppel Street, London WC1E 7HT, United Kingdom
3. Department of Infectious Disease Epidemiology, London School of Hygiene & Tropical Medicine, Keppel Street, London, United Kingdom
4. Livingstone Central Hospital, Livingstone, Zambia
5. Department of Infectious Diseases, Veterans Affairs Health Care System, California, USA
6. Department of Parasitology, Leiden University Medical Center, Leiden, The Netherlands
7. Department of Cell and Chemical Biology, Leiden University Medical Center, Leiden, The Netherlands.
8. Longhorn Vaccines and Diagnostics LLC, Bethesda, MD
9. Department of Global Health and Development, London School of Hygiene & Tropical Medicine, Keppel Street, London, United Kingdom
10. Wolfson Wellcome Biomedical Laboratories, Department of Zoology, Natural History Museum, Cromwell Road, London, United Kingdom
11. Department of Gynecology, University of Zambia, Lusaka, Zambia

***Corresponding author:**

Prof. Amaya Bustinduy

London School of Hygiene & Tropical Medicine

Keppel Street

WC1E 7HT- London, UK

Amaya.Bustinduy@lshtm.ac.uk

Word count: 3955

Figures:2

Tables:1

For peer review only

ABSTRACT

Introduction

Multiplathogen home-based self-sampling offers an opportunity to increase access to screening and treatment in endemic settings with high co-infection prevalence of sexually transmitted (HIV, *Trichomonas vaginalis (Tv),* human papillomavirus [HPV]) and non-sexually transmitted pathogens (*Schistosoma haematobium (Sh)).*Chronic co-infections may lead to disability (female genital schistosomiasis (FGS) and death (cervical cancer). The Zipime-Weka-Schista (Do self-testing sister!) aims to evaluate the validity, acceptability, uptake, impact and cost-effectiveness of *multi-pathogen* self-sampling for genital infections among women in Zambia.

Methods and Analysis

This is a longitudinal cohort study aiming to enrol 2,500 non-pregnant, sexually active and non-menstruating women aged 15-50 years from two districts in Zambia with two year follow up. During home visits, community health workers offer HIV and *Tv* self-testing and cervicovaginal self-swabs for (i) HPV by GeneXpert and, (ii) *Sh* DNA detection by conventional (PCR) and isothermal (RPA) molecular methods. *Schistosoma* ova and circulating anodic antigen are detected in urine. At a clinic follow-up midwifes perform same procedures and obtain hand-held colposcopic images. High-risk -HPV positive women are referred for a two-quadrant cervical biopsy according to age and HIV status. A *cost-effectiveness* analysis is conducted in parallel.

Ethics and dissemination

The University of Zambia Biomedical Research Ethics Committee (UNZABREC) approved the study in September 2021 (reference: 1858-2021), the London School of Hygiene and Tropical Medicine(LSHTM) (reference: 25258) and Ministry of Health and local superintendents. Written informed consent was obtained from all participants prior to enrolment. Identifiable data collected is stored securely and their confidentiality protected in accordance with the Data Protection Act 1998.

Keywords: Female genital schistosomiasis, *Schistosoma haematobium*, HIV, *Trichomonas vaginalis*, human papillomavirus (HPV), cervical cancer, self-sampling, acceptability, cost-effectiveness, hand-held colposcopy, RPA

For peer review only

For peer review only - http://bmjopen.bmj.com/site/about/guidelines.xhtml

Strenghts and Limitations

- The longitudinal design of the study will allow measurements of incidence and persistence of HPV in association with FGS and HIV and its joint contribution to cervical precancer and cancer.
- The one-stop home self-sampling (FGS, HR-HPV) and self-testing (HIV and Trichomonas)approach, if acceptable by participants, can become a scalable integrated screening strategy
- Cost-effectiveness data from the study will inform of community-based integration of FGS within the wider sexual and reproductive health screening in Zambia
- Recruitment may be limited by stigma pertaining to co-existing sexually transmitted infections
- Results may be delayed due to the limited availability of histopathology laboratories

For peer review only

INTRODUCTION

It is estimated that around 45 million women living in sub-Sahara Africa (SSA) are affected by Female genital schistosomiasis (FGS), a chronic, neglected and disabling gynaecological disorder associated with infertility, dyspareunia and symptoms mimicking sexually transmitted infections (STIs). (1, 2) FGS is difficult to diagnose and is part of the wider spectrum of urogenital disease caused by the waterborne parasite *Schistosoma haematobium* (*Sh*). In Zambia, reported FGS prevalence ranges from 33-75% of those with urinary schistosomiasis in endemic areas, suggesting a potentially high FGS burden. Data is not yet available on the FGS burden in the general population of Zambia. Furthermore, awareness of the disease and *non-sexual* mode of transmission is largely absent in endemic communities where information and education are urgently needed. (3)

Conventional FGS diagnosis is challenging as it relies on costly equipment and high-level specialized training is seldom available in resource-limited countries. Accurate estimation of disease burden is therefore hindered. However, the past decade has seen a dramatic increase in knowledge generation on many facets of FGS, and particularly on diagnostic strategies closer to the point-of-care. (4) Studies have shown acceptable sensitivity of genital swabs for the DNA detection of *Sh* from women's genital tract, potentially offering, for the first time, a scalable diagnostic approach for a parasitic disease that has been unacceptably neglected. (3, 4) Genital self-swabs are well accepted by participants and are known to increase uptake across settings. (5, 6)

There is growing evidence of an association of FGS with prevalent HIV, and suggestion of increased incidence and transmission of HIV in the presence of schistosome infections in women. (7) The lesions and tissue damage caused by FGS can provide an easy route for STI infections. (8) Further, women with schistosome worm infections and with FGS are potentially far more susceptible and at an increased risk of becoming HIV positive. (9) However, there is still a knowledge vacuum on the role of STIs in HIV acquisition in the presence of FGS, despite both STIs and *S.haematobium* infection being shown to be independently associated with HIV acquisition.(8, 10, 11)

For peer review only

For peer review only - http://bmjopen.bmj.com/site/about/guidelines.xhtml

There have been small studies suggesting an increased prevalence of high risk (HR-) HP, the etiological agent of cervical cancer, in women with FGS compared to those without ,(12) and higher odds of having an abnormal visual examination with acetic acid [VIA](13) and some suggestion of potential links between FGS and cervical cancer. (14, 15) In Zambia, cervical cancer is the most common female cancer, particularly in women aged 15 to 44 years(16, 17) and with high associated mortality (43.4 per 100,000 women). The lack of accessible screening and treatment programs, (18) and HPV vaccination contribute to the high cervical cancer incidence in these settings. (19) HPV-DNA based tests are recommended for primary screening of cervical cancer as part of the WHO 2021 cervical cancer screening and treatment guidelines (20), as better strategies than visual diagnosis. (21, 22) Despite validated self-sampling strategies for the detection of oncogenic or HR-HPV genotypes, community-based screening is not yet widely adopted alone or integrated with screening for other pathogens due to the high cost and infrastructure requirements of currently available tests. (23)

This study protocol describes the development and costing of a community-based comprehensive package for SRH screening in Zambia, including *sexually* (HPV, HIV, *Tv*) and *non-sexually* transmitted infections (*Sh)* in different endemicity settings with the long-term aim of preventing life threatening and disabling conditions such as cervical cancer, HIV and FGS. The overall justification of this study lies in the dearth of research exploring the feasibility of integrated screening and testing strategies of genital co-infections, and the epidemiological associations between them.(24-26)

METHODS AND DESIGN

Study design

The Zipime Weka Schista (Do self-testing sister!) study is a longitudinal cohort designed as a holistic approach to FGS detection at scale in the community. The study design will test the hypothesis that integration of home self-sampling for the screening of FGS in conjunction with HR-HPV and self-testing for HIV and STIs is an *diagnostically accurate*, *cost-effective* and *self-empowering* strategy that will increase the detection of cases and improve access to care for women of reproductive age in SSA.

The two main aims of the Schista study are:

For peer review only - http://bmjopen.bmj.com/site/about/guidelines.xhtml

1. To assess the performance of genital self-sampling, compared to healthcare provider sampling obtained for the detection of *Sh* by molecular testing (real-time PCR and Recombinase Polymerase Assay (RPA)) across two different *Sh* endemicity settings in Zambia, and
2. To assess the observed costs of integrating community based multiple pathogen genital self-sampling (*Sh*, HPV, STIs) and oral self-test (HIV) into one-stop home intervention and. project costs at scale.

Our secondary aims are to explore the epidemiological and immunological interrelationships between FGS and HPV as a potential risk factor for cervical cancer and expand knowledge on HIV acquisition in the presence of co- infections. We will also measure *acceptability* and *feasibility* of home-based multiple genital self-sampling and to measure HIV linkage to care and cervical cancer management after home self-sampling and testing. We will further aim to evaluate the effect of FGS and other STI on the diagnostic accuracy of visual inspection for detection of cervical precancer (CIN2+, CIN3+) and to describe the association of FGS, HPV, HIV and STIs with the presence concentration, diversity, and relative abundance of other key cervicovaginal microbiota and infections. We will also aim to consolidate testing for FGS and HPV into one field-applicable multiplex molecular assay and to recruit a sub-set of male household members of recruited participants screening male genital schistosomiasis.

Study setting and population

The study is being conducted in Lusaka and Southern provinces of Zambia. The specific study study areas are two sites in Kafue district with high (estimated *Sh* prevalence >70%) and moderate (estimated *Sh* prevalence >50%) prevalence and another site in Livingstone district with low prevalence (estimated *Sh* prevalence <10%). Each woman will have one home visit and one clinic visit at baseline with annual follow up for 2 years. Enrolment of eligible women began in January 2022 and is expected to be completed in May of 2024.

Inclusion/Exclusion criteria

All non-pregnant, sexually active women aged 15-50 years who are currently not menstruating and are able to provide consent are invited to take part in the study. In addition, women should be Zambian

For peer review only

For peer review only - http://bmjopen.bmj.com/site/about/guidelines.xhtml

residents of the endemic study area for at least one year.Women are excluded prior to their sexual debut ,pregnant or currently menstruating.

Sampling strategy and data collection procedures

Community-based cluster sampling was used to obtain a random sample of participants from the study communities. Communities and their households were mapped and each community was subdivided into blocks of approximately 50 households; each one would yield approximately 30 enrolled women. These community blocks were the primary sampling units, with all households in a selected block visited, and all eligible women aged 15-50 years invited to take part. Blocks were randomly selected using random number generation in Stata, and are being visited sequentially in the order that they were randomly selected. Participating women are assigned a unique identifier. Field data are entered using the London School of Hygiene and Tropical Medicine (LSHTM) ODK system and uploaded to a secure server in Zambia. The data are then transferred to a LSHTM secure server. The Chief Investigator is responsible for the data analysis with the support of the Department of Infectious Diseases Epidemiology at LSHTM.

Sample size and study power

A total of 2,500 women will be randomly selected from two districts (1,250 from each district). Based on previous assessments of FGS using PCR testing of genital samples from women in the study setting, we assume that 5-10% of genital samples from healthcare provider sampling will have *Sh* detected by molecular testing (5% in the lower endemicity area, 10% in the higher endemicity area). Taking clinic-based sampling as the gold standard, allowing for 10% loss to follow-up between the home and clinic sampling, and assuming that the diagnostic sensitivity and specificity of self-sampling compared to clinic-based sampling for the detection of *Sh* are 70% and 85%, respectively, we will be able to estimate the assumed target diagnostic sensitivity of 70% with a 95% CI of 62.3-76.6% overall, and the assumed target diagnostic specificity of 85% with a 95% CI of 83.4-86.5% (using the Clopper-Pearson formula to calculate an exact 95% confidence interval for this proportion). Considering individual districts, we will be able to estimate sensitivity of 70% with a 95% CI of 60.6-78.2% in the higher endemicity area and a 95% CI of 55.9-81.2%

For peer review only - http://bmjopen.bmj.com/site/about/guidelines.xhtml

in the lower endemicity area. Corresponding confidence intervals for specificity would be 83-87% in each area.

FIELD PROCEDURES

Community sensitization activities take place to inform target communities of the study aims and promote voluntary participation. Different support materials are used. Some of these include local drama groups, information brochures and an FGS manual edited by Zambart. Study community workers (SCWs) register expressions of interest and conduct home visits within the randomized zones to women that have expressed interest in participating in the study. All SCWs are females. Every participant has a total of three visits during the study: baseline, 12, and 24 months from recruitment. A schematic study flow is shown in **Figure 1**.

<< Insert Figure 1 here>>

Home visits

Home visits are conducted by SCWs who perform a demonstration of self-sampling, using a 3D model for illustration (supplementary figure 1). SCWs also obtain consent from participants and administer demographic and exposure questionnaires. After obtaining informed consent and completing questionnaires on demography, clinical symptoms and other relevant health and water contact information, as well as a Likert scale for acceptability, (27) the SCWs offer partcipants one genital self-swabs is stored in PrimeStore® MTM molecular transport media (donated by Longhorn Vaccines and Diagnostics LLC, Bethesda, MD) for for the detection of *Sh* by molecular methods (PCR and RPA) and the second genital swab is stored in Cytopreserve® for HR-HPV detection by Xpert. Participants are also offered two commercial self-tests: one oral (saliva) for HIV (28) and one vaginal self-swab for *Trichomonas vaginalis* (*Tv*). (29) For both self-tests, results are read by the SCW and given immediately. A confirmatory test is performed on site if there is a positive self-test for HIV. All HIV-positive women are referred to the local clinic for further management and linkage to care. For *Tv*, women are referred for treatment at the clinic visit. A urine sample is analysed via microscopy for *Sh* ova detection and circulating anodic antigen (CAA).(30)

For peer review only - http://bmjopen.bmj.com/site/about/guidelines.xhtml

Clinic visit

A follow up visit takes place at a local clinic. A trained healthcare provider (midwife) collects two cervicovaginal swabs and 5 ml of cervicovaginal lavage (CVL). Swabs are placed in PrimeStore® MTM molecular transport media (donated by Longhorn Vaccines and Diagnostics LLC, Bethesda, MD) and CVL are stored at -20 C on site for future detection of : 1) *Sh* DNA by real-time PCR as the reference assay for FGS; (31); 2) *Sh* DNA by Xpert® CT/NG (Chlamydia Trachomatis/Neisseria gonorrhoeae) (32) and studies of biomarkers and cervivovaginal microbiome. Approximately 5 ml of blood is collected by venipuncture in an EDTA tube for the detection of *Schistosoma* and STI serology. A hand-held colposcope, with an enhanced visual assessment (EVA) system is used to image the participant's cervix and vagina (MobileODT, Tel Aviv, Israel).Praziquantel and metronidazole are available free of charge for study partcipants with any positive result.(33)

Biopsy procedure

Women testing positive for any *high-risk HPV* by PCR (Xpert® HPV) during the home visit and depending on their age and HIV status *(see below*) are referred for colposcopy. Visual inspection using acetic acid and lugol's iodine *(VIA/VILI)* is performed by a trained gynecologist before biopsy of visible lesions. If no lesions are visible, two random biopsies are taken from normal areas including the transformation zone (at 12 and 6 o'clock), **Figure 2.** Cervical biopsies will be processed at the pathology laboratory at National Health laboratory Service, Johannesburg, South Africa and read using the 2-tier CIN classification system.

<< Insert Figure 2 here>>

HIV-negative women under the age of 25 and women living with HIV (WLHIV) under the age of 20 years have all procedures performed, including HPV testing in the community, but *they do not undergo biopsy in clinic* despite positive HR-HPV results. (34) This strategy follows ethical concerns of unnecessary biopsy and management of cervical lesions based on known HPV clearance and low risk of cervical precancer in young women. (35) For these women, there is annual longitudinal follow up for two years with repeated infection and clinical re-assessments.

For peer review only

For peer review only - http://bmjopen.bmj.com/site/about/guidelines.xhtml

Women diagnosed with cervical precancer and invasive cervical cancer:. If found to have histopathology verified CIN2+, participants are recalled for CIN2+ management. This visit is scheduled at the earliest convenient date once the result is known . In Zambia, all cancer patients are referred to the Cancer Diseases Hospital (CDH) based in Lusaka. Patients are filtered through the district and provincial hospitals serving as primary centres for cervical cancer screening countrywide before being referred to CDH for definitive treatment. Treatment at CDH is done according to the nationally and globally developed guidelines.(36)

LABORATORY PROCEDURES

The collection, storage and processing of all study samples is summarised in **Table 1**. Urine samples are processed for; 1) ***Urine parasitology*** Approximately 10mL (minimum) of urine is processed by centrifugation to detect *Sh* eggs by microscopy performed at the local government clinics in Kafue and Livingstone in Zambia by a trained technician and 2). ***CAA antigen assay.*** 2 mL of urine are stored at 4 ^{o}C and shipped to Lusaka and further to Leiden University Medical Center, The Netherlands. (37)

All genital samples (home/clinic) are processed for; 1) ***Detection of Schistosoma DNA*** using ITS-2 Real-time PCR processed at Zambart laboratories in Lusaka, Zambia.(31, 38) From the DNA already extracted for PCR additional well-validated qPCR assays will be used (39) to characterize the vaginal microbiota by quantifying key species and 2) ***Detection of Schistosoma DNA using RPA***. Up to 2 mL of fluid from vaginal lavage is stored at -20^{0}C and shipped to Lusaka. This assay is processed at Zambart laboratory in Zambia (40, 41) and 3) ***Inflammatory Markers***. Up to 3 mL of fluid from vaginal lavage is stored at -80 ^{o}C and shipped to Lusaka. (25, 31, 42)

<< Insert Table 1 here>>

Development of a multiplex assay for *S. haematobium* and HR-HPV testing

In a exploratory study we will aim to integrate into one molecular assay the RPA for *Sh* and HPV to increase accessibility and feasibility of molecular dual pathogen screenings after encouraging results on the validaty of the CVL RPA assay against standard real-time PCR,(43) which could be coupled with oncogenic HPV genotypes. (44)

For peer review only - http://bmjopen.bmj.com/site/about/guidelines.xhtml

Qualitative assessment

In-Depth feasibility and acceptability of integrated sampling, testing and treatment

Previous work has documented the acceptability of performing multiple genital swabs at a single encounter .(27) For the current proposal, in-depth cognitive interviews will be delivered to a subset of participants to evaluate the acceptability of the multi-pathogen self-swabbing strategy (three genital swabs and one oral swab). (45)

STATISTICAL ANALYSIS

To assess the performance of genital self-sampling compared to healthcare provider sampling for the detection of *Sh,* sensitivity, specificity, PPV and NPV will be calculated together with 95% confidence intervals. Results will be presented overall and stratified by geographic setting. To explore associations of baseline FGS with prevalence of cervical precancer at baseline and cervical precancer incidence at 12 and 24 months, logistic regression will be used to estimate odds ratios. Associations between baseline FGS and HR-HPV persistence at 12 months will be estimated with generalized estimating equation to account for multiple HR-HPV infection and multiple infection states (persistence and clearance).(46) The association of FGS with HIV acquisition will be assessed using Poisson regression, controlling for key confounders.

Other outcome measures obtained in the study will include 1)HIV incidence rate, HR-HPV clearance and persistence, prevalence and incidence of cervical precancer (histology confirmed CIN2 or CIN3+), measures of association between different exposures (reproductive tract infections); 2) Acceptability of multiple genital self-sampling interventions ; 3) Rates of ART initiation, continuation of treatment and access to care ; 4) Cervical precancer diagnosis and treatment, testing and management of STIs. 4) Presence or absence of cervicovaginal microbiota in women with FGS, HPV and STIs. Expressed as relative abundance (percentage abundance of each species), genomic concentration per microorganism (genome equivalents per mL); 5) Sensitivity, specificity, PPV, NPV of visual inspection of the cervix according to FGS status and Integrated molecular field-applicable testing platform for *Sh* and HR-HPV.

For peer review only

For peer review only - http://bmjopen.bmj.com/site/about/guidelines.xhtml

Quality control

Several procedures have been employed to ensure the quality of the study data, maximizing the validity and reliability of the data obtained and of the evaluation of the results: These include the following (a) 10 % of all samples will be reviewed blindly by experts at different institutions; parasitology (Liverpool School of Tropical Medicine, Liverpool, UK), PCR (Natural History Museum, London, UK), colposcopic images (University of Zambia, Lusaka, Zambia)

Economic analysis

A full economic costing from a provider's and patient's perspective is ongoing alongside data collection. Cost data is collected combining a bottom-up and top-down approach following established costing guidelines, (47) and STI self-testing economic evaluations in the.region. This allows us to estimate the total intervention cost, and unit costs per: woman screened, FGS infection detected, per case of CIN2+ detected, per person linked to care, per person successfully treated overall and per pathogen identified. We will model the costs across a set of scale-up scenarios to understand the budget implications of introducing widescale self-screening. Populating a health economics decision model with the study outcomes, we will identify cost thresholds under which joint self-sampling for FGS, HPV, STIs and HIV is cost-effective. This will be relevant for informing the maximum threshold diagnostic kit price based on its potential cost-effectiveness.(48) MoH and policy makers will be informed on the most cost-effective screening strategy to implement for FGS screening. This study constitutes the first economic evaluation of FGS.

Patient and Public involvement

Participants who are being recruited to this study did not take part in the design or implementation of the study. They are however deeply involved finding solutions for better follow up through focus groups discussions and mobilisation activities in the community.

DISCUSSION

Sexual and reproductive health is a critical area of development highlighted in the United Nations Sustainable Development Goal 3. There is an estimated 40 million women unknowingly living with FGS,

For peer review only - http://bmjopen.bmj.com/site/about/guidelines.xhtml

with dire consequences for their sexual and reproductive lives which include infertility, pain with sexual relations, ectopic pregnancies and increased risk of HIV acquisition and cervical precancer development, mostly in countries with high prevalence of STIs. This study will provide high quality evidence on the validity, acceptability, uptake and costs of integrating community based multiple-pathogen *genital self-sampling* (*Sh*, HR-HPV, STIs) and *oral self-test (*HIV) into *one-stop intervention*. Findings will help inform policy and practice at local and regional levels. Importantly, this study brings together existing and new partnerships for a multidisciplinary investigative approach, to explore the value of integration of reproductive health screening practices through self-sampling and testing. The baseline results will provide further validation of self-swabs as an acceptable sampling strategy for FGS diagnosis in a larger sample population and with a wider scope than previously performed. Its longitudinal approach will also provide an allied impact assessment on the incidence of cervical precancer, HIV and STIs and early HIV detection and access to treatment for the wider improvement of women's SRH in Zambia. This is important within the growing evidence of a link between FGS and cervical cancer. (15) The clear interplay between genital infections highlights the urgent need to bolster programmes that jointly integrate strategies to screen and detect multiple genital infections with simple, affordable and as close to the point-of-care as possible.

Genital self-sampling and self-testing through community based programmes are widely accepted strategies for the detection of devastating diseases like HIV in both adults and adolescents. (8, 31, 49) HIV self-testing programmes have been shown to be cost-effective. Genital self-sampling has enhanced access to health services among hard-to-reach populations such as young people, (31) and those who do not regularly access health screening services. (1, 50) It also empowers women and protect their privacy since self-swabs can be performed at home or in other private places and increases compliance with testing. A high proportion of women, including those from resource-limited settings have been found to prefer vaginal specimen self-collection (51) compared with clinic-based sampling. In addition to acceptability, two other factors make genital self-sampling advantageous; 1) the availability of vaginal self-sampling is effective for improving participation in specific SRH screening programmes and 2) the sensitivity of PCR-based assays on self-collected specimens compares favourably with physician-performed sampling.(1)

For peer review only - http://bmjopen.bmj.com/site/about/guidelines.xhtml

The synergistic approach proposed in this study is justified by the known and suspected interactions of the different genital co-infections. FGS is associated with genital inflammation, friable blood vessels and contact bleeding. (52-54) The disruptions of the mucosal surface in FGS may place women at risk for HIV, as has been found for several STIs. (55-57) Women with urogenital schistosomiasis had a 3-4-fold higher odds of prevalent HIV in cross-sectional studies in Zimbabwe, Tanzania and Mozambique. (58-62) However, FGS as a risk factor for HIV is not yet integrated in ongoing screening programmes, including HIV self-testing strategies. (63-65)

Current FGS diagnostic strategies are limited because they require expertise and equipment that may not be readily available in low income settings. Several diagnostics will be tested in this study to provide 'real-world' data on the scalability of the different approaches. Hand-held colposcopy as a low-cost device has been used in both cervical cancer screening and FGS studies. (4, 66) However, expert review is still required for the identification of characteristic FGS lesions and inter-rater correlation is poor. (67) This study will enhance diagnostics by applying multipathogen DNA testing with conventional molecular assays that are field deployable, such as the RPA (32, 40) Following on the positive correlation between RPA assay from CVL against standard real-time PCR, (68) in the Schista study we will aim to develop a multiplex RPA assay for *Sh* and HR-HPV to increase accessibility and feasibility of dual pathogen molecular screenings.

To make interventions ultimately sustainable these need to be cost-effective. In this study we aim to assess for the first time the observed costs of integrating community based multiple-pathogen genital self-sampling (*Sh*, HPV, STIs) and oral self-test (HIV) in a *one-stop* intervention and project costs at scale for the justification of the diagnostic kit price based on its impact on disease prevalence. (26)

To conclude, the Schista study has a number of potential benefits at both individual and community levels including diagnosis and treatment of previously undiagnosed *S. haematobium* infection as well as diagnosis, treatment and linkage to care for cervical cancer, HIV and STIs. Multi-pathogen self-sampling and testing constitutes an unmissable opportunity to offer potentially life-changing health services closer to the users.(6)

.

For peer review only - http://bmjopen.bmj.com/site/about/guidelines.xhtml
For peer review only

DECLARATIONS

Competing interest statement

The authors declare they have no competing interests.

Data availability statement

Data will be available upon request on LSHTM Data Compass

Funding statement

This study is funded through a UKRI Future Leaders Fellowship (MR/T041900/1) awarded to Prof. AL Bustinduy. Prof E Webb and Prof R Hayes received funding from MRC Grant Reference MR/K012126/1. This award is jointly funded by the UK Medical Research Council (MRC) and the UK Department for International Development (DFID) under the MRC/DFID Concordat agreement and is also part of the EDCTP2 programmme supported by the European Union. Funders had no role in study design, data collection and analysis, decision to publish, or preparation of the manuscript.

Authors' Contributions

AB originally conceived and designed the study in consultation with PM, HA, BW, IH, FTP and RH. KS wrote the first draft of this manuscript. BW, HK and AS advised on primary and some secondary objectives, FTP and OL conceived the economic analysis, EW and NK helped design the statistical framework of the study and contributed to all drafts of the manuscript. BK, BW, LVL, JF, GVD and PC contributed to the laboratory component of the study. RN VB and BVcontributed to the design of secondary objectives. All authors read and approved the final manuscript.

Acknowledgements

We would like to thank and acknowledge all the participants, Schista! study fieldworkers and midwives. and the wider Schista! study team who contributed towards the design of the study. Also, our partners in Zambia including the Ministry of Health and District Health Management Teams; the administrative and support teams at Zambart and LSHTM.

REFERENCES

1. Kjetland EF, Kurewa EN, Mduluza T, et al. The first community-based report on the effect of genital Schistosoma haematobium infection on female fertility. *Fertil Steril*. 2010;94:1551-1553.
2. Bustinduy AL, Randriansolo B, Sturt AS, et al. Chapter One - An update on female and male genital schistosomiasis and a call to integrate efforts to escalate diagnosis, treatment and awareness in endemic and non-endemic settings: The time is now. In: Rollinson D, Stothard R, eds. *Advances in Parasitology*: Academic Press; 2022:1-44.

For peer review only

For peer review only - http://bmjopen.bmj.com/site/about/guidelines.xhtml

3. Hotez PJ, Harrison W, Fenwick A, et al. Female genital schistosomiasis and HIV/AIDS: Reversing the neglect of girls and women. *PLoS Negl Trop Dis*. 2019;13:e0007025.
4. Sturt AS, Webb EL, Phiri CR, et al. Genital self-sampling compared with cervicovaginal lavage for the diagnosis of female genital schistosomiasis in Zambian women: The BILHIV study. *PLoS Negl Trop Dis*. 2020;14:e0008337.
5. Mbatha JN, Galappaththi-Arachchige HN, Mtshali A, et al. Self-sampling for human papillomavirus testing among rural young women of KwaZulu-Natal, South Africa. *BMC Res Notes*. 2017;10:702.
6. Francis SC, Mthiyane TN, Baisley K, et al. Prevalence of sexually transmitted infections among young people in South Africa: A nested survey in a health and demographic surveillance site. *PLoS Med*. 2018;15:e1002512.
7. Wall KM, Kilembe W, Vwalika B, et al. Schistosomiasis is associated with incident HIV transmission and death in Zambia. *PLoS Negl Trop Dis*. 2018;12:e0006902.
8. Kjetland EF, Ndhlovu PD, Gomo E, et al. Association between genital schistosomiasis and HIV in rural Zimbabwean women. *AIDS*. 2006;20:593-600.
9. Downs JA, Dupnik KM, van Dam GJ, et al. Effects of schistosomiasis on susceptibility to HIV-1 infection and HIV-1 viral load at HIV-1 seroconversion: A nested case-control study. *PLoS Negl Trop Dis*. 2017;11:e0005968.
10. Downs JA, Mguta C, Kaatano GM, et al. Urogenital schistosomiasis in women of reproductive age in Tanzania's Lake Victoria region. *Am J Trop Med Hyg*. 2011;84:364-369.
11. Wall KM, Kilembe W, Vwalika B, et al. Schistosomiasis is associated with incident HIV transmission and death in Zambia. *PLOS Neglected Tropical Diseases*. 2018;12:e0006902.
12. Kjetland EF, Ndhlovu PD, Mduluza T, et al. The effects of genital schistosoma haematobium on human papillomavirus and the development of cervical neoplasia after five years in a Zimbabwean population. *Eur J Gynaecol Oncol*. 2010;31:169-173.
13. Rafferty H, Sturt AS, Phiri CR, et al. Association between cervical dysplasia and female genital schistosomiasis diagnosed by genital PCR in Zambian women. *BMC Infect Dis*. 2021;21:691.
14. Vlassoff C, Arogundade K, Patel K, Jacobson J, Gyapong M, Krentel A. Improving the Response of Health Systems to Female Genital Schistosomiasis in Endemic Countries through a Gender-Sensitive Human Rights-Based Framework. *Diseases*. 2022;10:125.
15. Engels D, Hotez PJ, Ducker C, et al. Integration of prevention and control measures for female genital schistosomiasis, HIV and cervical cancer. *Bull World Health Organ*. 2020;98:615-624.
16. Bray F, Ferlay J, Soerjomataram I, Siegel RL, Torre LA, Jemal A. Global cancer statistics 2018: GLOBOCAN estimates of incidence and mortality worldwide for 36 cancers in 185 countries. *CA Cancer J Clin*. 2018;68:394-424.
17. Bruni L AG, Serrano B, Mena M, Gómez D, Muñoz J, Bosch FX, de Sanjosé S. . Human Papillomavirus and Related Diseases in Zambia. Summary Report 17 June 2019. 2019. Accessed 15th October, 2019.
18. Bruni L, Serrano B, Roura E, et al. Cervical cancer screening programmes and age-specific coverage estimates for 202 countries and territories worldwide: a review and synthetic analysis. *Lancet Glob Health*. 2022;10:e1115-e1127.
19. Bruni L, Saura-Lázaro A, Montoliu A, et al. HPV vaccination introduction worldwide and WHO and UNICEF estimates of national HPV immunization coverage 2010-2019. *Prev Med*. 2021;144:106399.
20. World Health Organization (2021). WHO guideline for screening and treatment of cervical pre-cancer lesions for cervical cancer prevention, 2nd ed. World Health Organization. https://apps.who.int/iris/handle/10665/342365. License: CC BY-NC-SA 3.0 IGO.
21. Arbyn M, Sankaranarayanan R, Muwonge R, et al. Pooled analysis of the accuracy of five cervical cancer screening tests assessed in eleven studies in Africa and India. *Int J Cancer*. 2008;123:153-160.
22. Arbyn M, Simon M, Peeters E, et al. 2020 list of human papillomavirus assays suitable for primary cervical cancer screening. *Clin Microbiol Infect*. 2021;27:1083-1095.
23. Arbyn M, Smith SB, Temin S, et al. Detecting cervical precancer and reaching underscreened women by using HPV testing on self samples: updated meta-analyses. *BMJ*. 2018;363:k4823.

For peer review only - http://bmjopen.bmj.com/site/about/guidelines.xhtml

24. Mezei AK, Pedersen HN, Sy S, et al. Community-based HPV self-collection versus visual inspection with acetic acid in Uganda: a cost-effectiveness analysis of the ASPIRE trial. *BMJ Open*. 2018;8:e020484.
25. Lunny C, Taylor D, Hoang L, et al. Self-Collected versus Clinician-Collected Sampling for Chlamydia and Gonorrhea Screening: A Systemic Review and Meta-Analysis. *PLoS One*. 2015;10:e0132776.
26. Mangenah C, Mwenge L, Sande L, et al. Economic cost analysis of door-to-door community-based distribution of HIV self-test kits in Malawi, Zambia and Zimbabwe. *J Int AIDS Soc*. 2019;22 Suppl 1:e25255.
27. Rutty Phiri C, Sturt AS, Webb EL, et al. Acceptability and feasibility of genital self-sampling for the diagnosis of female genital schistosomiasis: a cross-sectional study in Zambia. *Wellcome Open Res*. 2020;5:61.
28. Zachary D, Mwenge L, Muyoyeta M, et al. Field comparison of OraQuick ADVANCE Rapid HIV-1/2 antibody test and two blood-based rapid HIV antibody tests in Zambia. *BMC Infect Dis*. 2012;12:183.
29. Campbell L, Woods V, Lloyd T, Elsayed S, Church DL. Evaluation of the OSOM Trichomonas rapid test versus wet preparation examination for detection of Trichomonas vaginalis vaginitis in specimens from women with a low prevalence of infection. *J Clin Microbiol*. 2008;46:3467-3469.
30. van Dam GJ, de Dood CJ, Lewis M, et al. A robust dry reagent lateral flow assay for diagnosis of active schistosomiasis by detection of Schistosoma circulating anodic antigen. *Exp Parasitol*. 2013;135:274-282.
31. Kjetland EF, Hove RJ, Gomo E, et al. Schistosomiasis PCR in vaginal lavage as an indicator of genital Schistosoma haematobium infection in rural Zimbabwean women. *Am J Trop Med Hyg*. 2009;81:1050-1055.
32. Garrett N, Mitchev N, Osman F, et al. Diagnostic accuracy of the Xpert CT/NG and OSOM Trichomonas Rapid assays for point-of-care STI testing among young women in South Africa: a cross-sectional study. *BMJ Open*. 2019;9:e026888.
33. Ministry of Health. National cancer control strategic plan 2016-2021. Lusaka: Zambia 2017. Available at: https://www.iccp-portal.org/system/files/plans/NCCSP%20Final%20Version%20Zambia.pdf,. Accessed 5th October, 2022.
34. Parham GP, Mwanahamuntu MH, Kapambwe S, et al. Population-level scale-up of cervical cancer prevention services in a low-resource setting: development, implementation, and evaluation of the cervical cancer prevention program in Zambia. *PLoS One*. 2015;10:e0122169.
35. Woodman CB, Collins S, Winter H, et al. Natural history of cervical human papillomavirus infection in young women: a longitudinal cohort study. *Lancet*. 2001;357:1831-1836.
36. Mumba JM, Kasonka L, Owiti OB, et al. Cervical cancer diagnosis and treatment delays in the developing world: Evidence from a hospital-based study in Zambia. *Gynecol Oncol Rep*. 2021;37:100784.
37. Corstjens PL, van Lieshout L, Zuiderwijk M, et al. Up-converting phosphor technology-based lateral flow assay for detection of Schistosoma circulating anodic antigen in serum. *J Clin Microbiol*. 2008;46:171-176.
38. Obeng BB, Aryeetey YA, de Dood CJ, et al. Application of a circulating-cathodic-antigen (CCA) strip test and real-time PCR, in comparison with microscopy, for the detection of Schistosoma haematobium in urine samples from Ghana. *Ann Trop Med Parasitol*. 2008;102:625-633.
39. Jespers V, van de Wijgert J, Cools P, et al. The significance of Lactobacillus crispatus and L. vaginalis for vaginal health and the negative effect of recent sex: a cross-sectional descriptive study across groups of African women. *BMC Infect Dis*. 2015;15:115.
40. Rosser A, Rollinson D, Forrest M, Webster BL. Isothermal Recombinase Polymerase amplification (RPA) of Schistosoma haematobium DNA and oligochromatographic lateral flow detection. *Parasit Vectors*. 2015;8:446.
41. Utzinger J, Becker SL, van Lieshout L, van Dam GJ, Knopp S. New diagnostic tools in schistosomiasis. *Clin Microbiol Infect*. 2015;21:529-542.
42. Sturt AS, Webb EL, Patterson C, et al. Cervicovaginal Immune Activation in Zambian Women With Female Genital Schistosomiasis. *Front Immunol*. 2021;12:620657.

For peer review only - http://bmjopen.bmj.com/site/about/guidelines.xhtml

43. Archer J, Patwary FK, Sturt AS, et al. Validation of the isothermal Schistosoma haematobium Recombinase Polymerase Amplification (RPA) assay, coupled with simplified sample preparation, for diagnosing female genital schistosomiasis using cervicovaginal lavage and vaginal self-swab samples. *PLOS Neglected Tropical Diseases*. 2022;16:e0010276.
44. Ma B, Fang J, Wang Y, et al. Isothermal Method of a Recombinase Polymerase Amplification Assay for the Detection of Most Common High-Risk Human Papillomavirus Type 16 and Type 18 DNA. *Clin Lab*. 2017;63:27-38.
45. Simwinga M KM, Dacombe RJ, Kayira L, Muzumara A, Johnson CC, et al. Ability to understand and correctly follow HIV self-test kit instructions for use: applying the cognitive interview technique in Malawi and Zambia. *J Int AIDS Soc*.22 Suppl 1:e25253.
46. Xue X, Gange SJ, Zhong Y, et al. Marginal and Mixed-Effects Models in the Analysis of Human Papillomavirus Natural History Data. *Cancer Epidemiology, Biomarkers & Prevention*. 2010;19:159 - 169.
47. Vassall A, Sweeney, S., Kahn, J., Gomez, G. B., Bollinger, L., Marseille, E., … & Levin, C, . Reference Case for Estimating the Costs of Global Health Services and Interventions. Global Health Cost Consortium. 2017. Available at: https://ghcosting.org/pages/standards/reference_case Accessed 15th March, 2022.
48. Terris-Prestholt F, Foss AM, Cox AP, et al. Cost-effectiveness of tenofovir gel in urban South Africa: model projections of HIV impact and threshold product prices. *BMC Infect Dis*. 2014;14:14.
49. Kjetland EF, Poggensee G, Helling-Giese G, et al. Female genital schistosomiasis due to Schistosoma haematobium. Clinical and parasitological findings in women in rural Malawi. *Acta Trop*. 1996;62:239-255.
50. Pillay P, Downs JA, Changalucha JM, et al. Detection of Schistosoma DNA in genital specimens and urine: A comparison between five female African study populations originating from S. haematobium and/or S. mansoni endemic areas. *Acta Tropica*. 2020;204:105363.
51. Helling-Giese G, Kjetland EF, Gundersen SG, et al. Schistosomiasis in women: manifestations in the upper reproductive tract. *Acta Tropica*. 1996;62:225-238.
52. Yeh HY, Mitchell PD. Ancient Human Parasites in Ethnic Chinese Populations. *The Korean journal of parasitology*. 2016;54:565-572.
53. Barakat RM. Epidemiology of Schistosomiasis in Egypt: Travel through Time: Review. *Journal of advanced research*. 2013;4:425-432.
54. Madden F. A case of Bilharzia of the vagina. *Lancet*. 1899;1:1716-1710.
55. Kaul R, Pettengell C, Sheth PM, et al. The genital tract immune milieu: an important determinant of HIV susceptibility and secondary transmission. *J Reprod Immunol*. 2008;77:32-40.
56. Masson L, Passmore JA, Liebenberg LJ, et al. Genital inflammation and the risk of HIV acquisition in women. *Clin Infect Dis*. 2015;61:260-269.
57. Kjetland EF, Hegertun IE, Baay MF, Onsrud M, Ndhlovu PD, Taylor M. Genital schistosomiasis and its unacknowledged role on HIV transmission in the STD intervention studies. *International journal of STD & AIDS*. 2014;25:705-715.
58. Kjetland EF, Leutscher PD, Ndhlovu PD. A review of female genital schistosomiasis. *Trends Parasitol*. 2012;28:58-65.
59. Downs JA, Mguta C, Kaatano GM, et al. Urogenital schistosomiasis in women of reproductive age in Tanzania's Lake Victoria region. *Am J Trop Med Hyg*. 2011;84:364-369.
60. Hotez PJ, Fenwick A, Kjetland EF. Africa's 32 cents solution for HIV/AIDS. *PLoS Negl Trop Dis*. 2009;3:e430.
61. Brodish PH, Singh K. Association between *Schistosoma haematobium* exposure and Human Immunodeficiency Virus infection among females in Mozambique. *Am J Trop Med Hyg*. 2016;94:1040-1044.
62. Ndeffo Mbah ML, Poolman EM, Drain PK, Coffee MP, van de Werf MJ, Galvani AP. HIV and Schistosoma haematobium prevalences correlate in sub-Saharan Africa. *Tropical Medicine and International Health*. 2013.

For peer review only - http://bmjopen.bmj.com/site/about/guidelines.xhtml

63. Berge ST, Kabatereine NB, Gundersen SG, et al. Generic praziquantel in South Africa: The necessity for policy change to avail cheap, safe and efficacious schistosomiasis drugs to the poor, rural population *Southern Afr J Epidemiol Infect*. 2011;26:22-25.

64. Lothe A. Treating Bilharzia among High School Pupils. A study of opportunities and constraints for treating Bilharzia among high school pupils in Ugu district, South Africa. *Faculty of Economics and Social Sciences*. Development Management: Agder University College; 2012:112.

65. Ndhlovu PD, Mduluza T, Kjetland EF, et al. Prevalence of urinary schistosomiasis and HIV in females living in a rural community of Zimbabwe: does age matter? *Transactions of the Royal Society of Tropical Medicine and Hygiene*. 2007;101:433-438.

66. Taghavi K, Moono M, Mwanahamuntu M, et al. Screening test accuracy to improve detection of precancerous lesions of the cervix in women living with HIV: a study protocol. *BMJ Open*. 2020;10:e037955.

67. Sturt A, Bristowe H, Webb E, et al. Visual diagnosis of female genital schistosomiasis in Zambian women from hand-held colposcopy: agreement of expert image review. *Wellcome Open Res*. 2023;8:14.

68. Archer J, Patwary FK, Sturt AS, et al. Validation of the isothermal Schistosoma haematobium Recombinase Polymerase Amplification (RPA) assay, coupled with simplified sample preparation, for diagnosing female genital schistosomiasis using cervicovaginal lavage and vaginal self-swab samples. *PLoS Negl Trop Dis*. 2022;16:e0010276.

Figure legends

Figure 1. Schista study flow

Figure 2. Flowchart for cervical precancer determination in HIV negative an women living with HIV

Supplementary Information

Supplemental figure:3D models for the teaching of self sa

Table 1: Sample storage and processing for the Schista study.

For peer review only - http://bmjopen.bmj.com/site/about/guidelines.xhtml

*High risk HPV by self-collected swab only

Sample	Location	Storage	Sample Processing	Location of processing
**Self-collected swab Provider-collected swab**	Home	Room temperature (Stored in Primestore media®)	Sh DNA (PCR, RPA)<br><br>GeneXpert- High Risk HPV DNA*	Zambart (Zambia)
**Urine for microscopy**	Home	Room temperature	Sh eggs<br>Urine haemastix	District Laboratory (Kafue, Livingstone, Zambia)
**Urine stored for CAA**	Clinic	4 °C	Circulating Anodic Antigen	Leiden University Medical Centre (The Netherlands)
**Cervicovaginal lavage**	Clinic	-20 °C	Sh DNA (PCR, RPA)<br><br>GeneXpert- CT/NG<br><br>Microbiota	Zambart (Zambia)
**Blood for serology**	Clinic	-20 °C	Sh, STI Antibody	Zambart (Zambia)
**Colposcopy images**	Clinic	Encrypted server	Expert Review Computer Analysis	Zambia<br>London School of Hygien & Tropical Medicine (UK)

List of Abbreviations

For peer review only - http://bmjopen.bmj.com/site/about/guidelines.xhtml

AE	Adverse Event
EDC	Electronic Data Capture
FGS	Female Genital Schistosomiasis
CAA	Circulating anodic antigen
CIN	Cervical intraepithelial neoplasia
ICC	Invasive Cervical Cancer
LLETZ	Large loop excision of the transformation zone
CW	Community workers
HR-HPV	High Risk Human Papilloma Virus
ODK	Open Data Kit
PCR	Polymerase Chain Reaction
RPA	Recombinase Polymerase Amplification
SCW	Schista Community Worker
SRH	Sexual and Reproductive Health
Sh	*Schistosoma haematobium*
SSA	Sub-Saharan Africa
STI	Sexually transmitted infections
Tv	*Trichomonas vaginalis*
VIA	Visual inspection with acetic acid

For peer review only - http://bmjopen.bmj.com/site/about/guidelines.xhtml

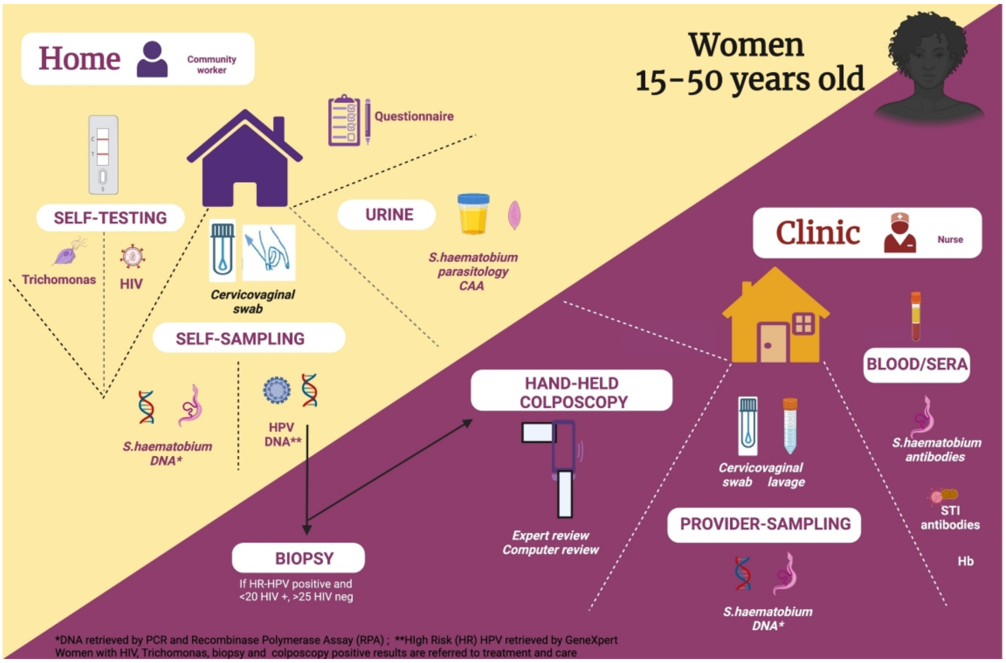

Figure 1. Schista study flow

251x165mm (300 x 300 DPI)

Figure 2 . Flowchart for cervical precancer determination in HIV negative and women living with HIV

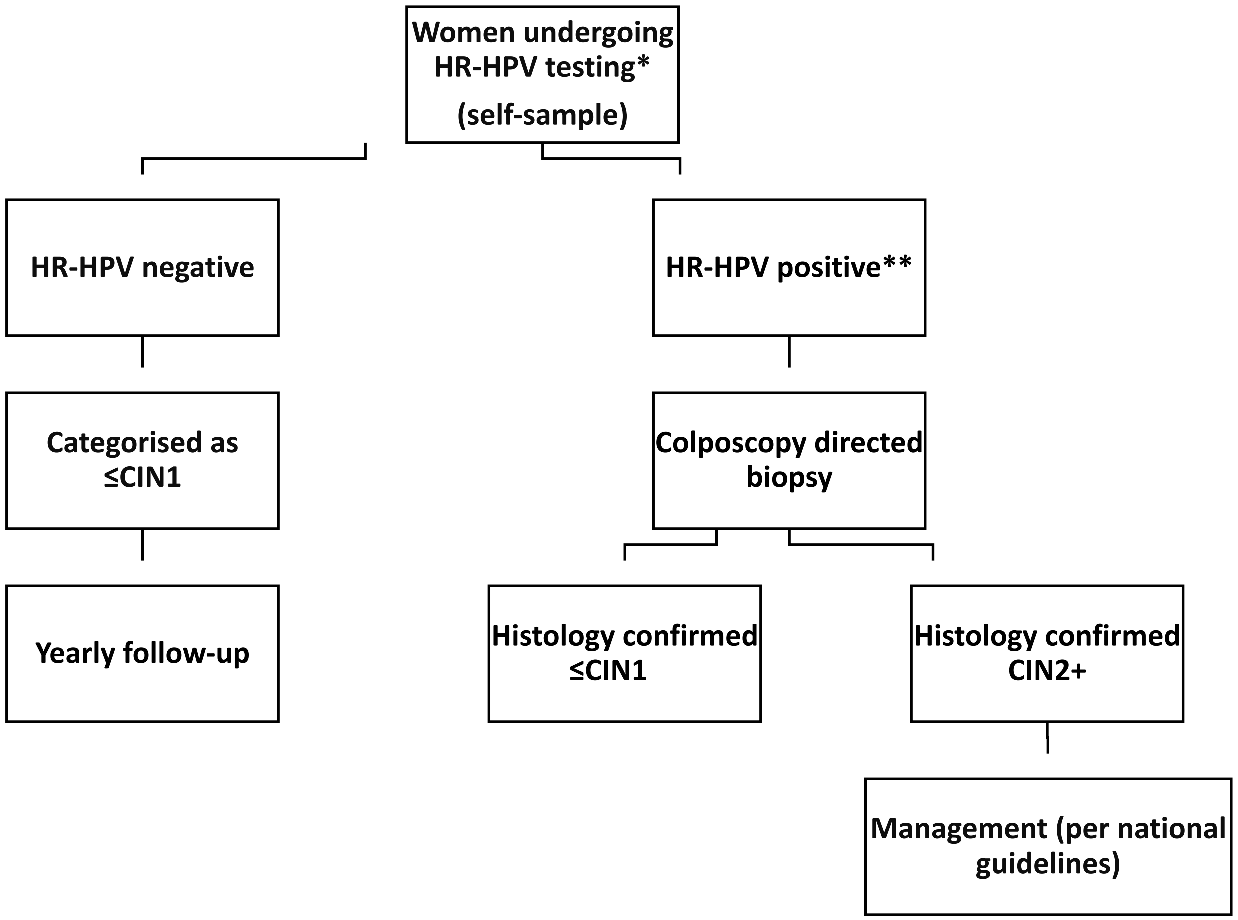

**HR-HPV detected using Xpert; **HR-HPV positive WLHIV aged 20 years and older and HIV-negative women aged 25 years and older only.*

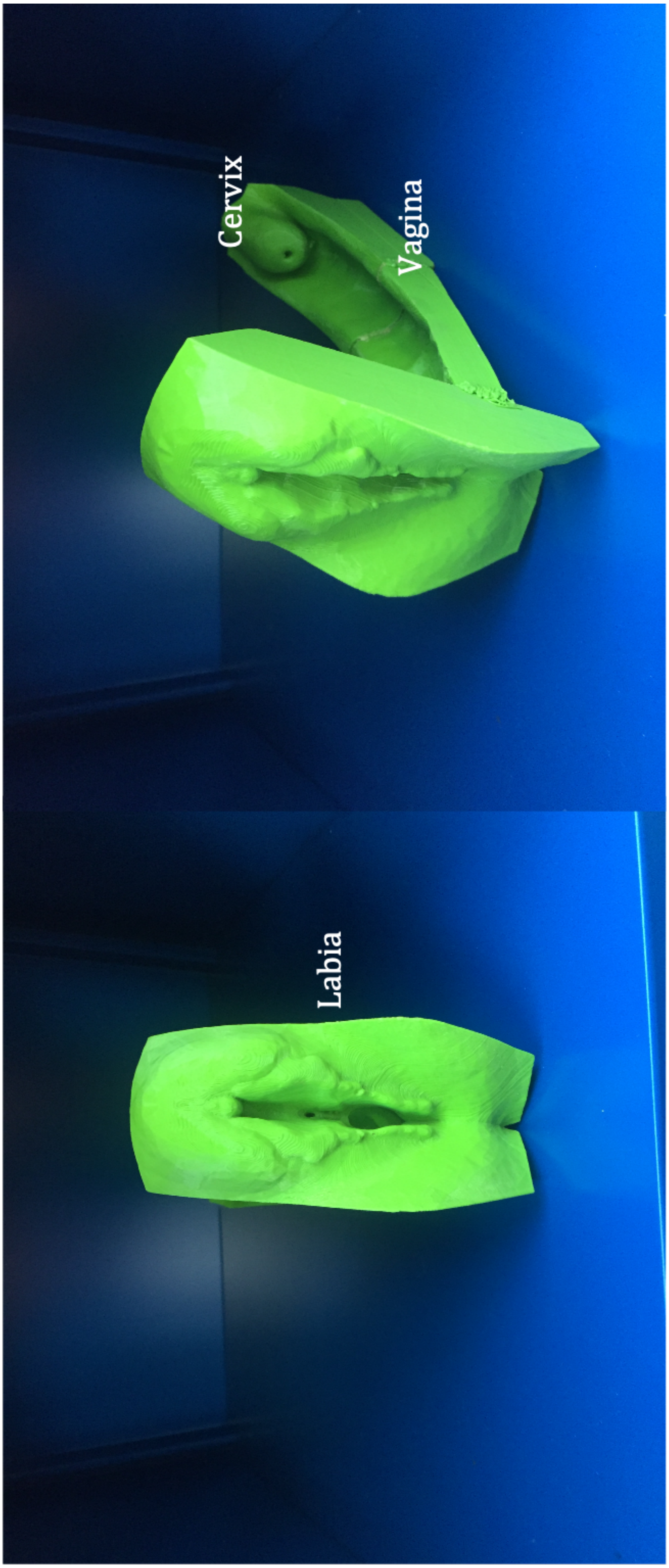

Supplementary figure 1: 3 D models for the teaching of self-sampling

BMJ Open

The Zipime-Weka-Schista study protocol: a longitudinal cohort study and economic evaluation of an integrated home-based approach for genital multi-pathogen screening in women, including female genital schistosomiasis, Human Papilloma Virus, Trichomonas and HIV in Zambia

Journal:	*BMJ Open*
Manuscript ID	bmjopen-2023-080395.R2
Article Type:	Protocol
Date Submitted by the Author:	01-May-2024
Complete List of Authors:	Shanaube, Kwame; Zambart<br>Ndubani, Rhoda; Zambart<br>Kelly, Helen; London School of Hygiene and Tropical Medicine, Clinical Research Department<br>Webb, Emily; London School of Hygiene and Tropical Medicine, Faculty of Infectious and Tropical Diseases<br>Mayaud, Philippe; London School of Hygiene & Tropical Medicine Faculty of Infectious and Tropical Diseases, Clinical Research<br>Lamberti, Olimpia; London School of Hygiene and Tropical Medicine, Clinical Research Department<br>Fitzpatrick, Jennifer; Zambart<br>Kasese, Nkatya; Zambart<br>Sturt, Amy; Veterans Affairs Health Care System, Department of Infectious Diseases<br>Van Lieshout, Lisette; Leiden University Medical Center<br>Van Dam, Govert; Leiden University Medical Center<br>Corstjens, Paul; Leiden University Medical Center, Department of Cell and Chemical Biology<br>Kosloff, Barry; Zambart<br>Bond, Virginia; London School of Hygiene & Tropical Medicine Centre of Global Change and Health<br>Hayes, Richard; London School of Hygiene and Tropical Medicine, Epidemiology and Population Health<br>Terris-Prestholt, Fern; London School of Hygiene & Tropical Medicine<br>Webster, Bonnie; Natural History Museum<br>Vwalika, Bellington; University of Zambia School of Medicine, Department of Obstetrics & Gynaecology<br>Hansingo, Isaiah; Livingstone Central Hospital, Gynecology<br>Ayles, Helen; London School of Hygiene & Tropical Medicine; Zambart<br>Bustinduy, Amaya; London School of Hygiene and Tropical Medicine, Clinical Research; London School of Hygiene & Tropical Medicine
**Primary Subject Heading**:	Global health
Secondary Subject Heading:	Sexual health

Keywords:	Adolescent, PUBLIC HEALTH, SEXUAL MEDICINE, Molecular diagnostics < INFECTIOUS DISEASES, PARASITOLOGY, Colposcopy < GYNAECOLOGY

SCHOLARONE™
Manuscripts

The Zipime-Weka-Schista study protocol: a longitudinal cohort study and economic evaluation of an integrated home-based approach for genital multi-pathogen screening in women, including female genital schistosomiasis, Human Papilloma Virus, Trichomonas and HIV in Zambia

*Kwame Shanaube[1], Rhoda Ndubani[1], Helen Kelly[2], Emily L. Webb[3], Philippe Mayaud[2], Olimpia Lamberti[2], Jennifer Fitzpatrick[1], Nkatya Kasese[1], Amy Sturt[5], Lisette van Lieshout[6], Govert J. van Dam[6], Paul L.A.M. Corstjens[7], Barry Kosloff[18], Virginia Bond[1,2], Richard Hayes[3], Fern Terris-Prestholt[9], Bonnie Webster[10], Bellington Vwalika[11], Isaiah Hansingo[4], Helen Ayles[1,2], Amaya L. Bustinduy[2]

1. Zambart, Lusaka, Zambia
2. Department of Clinical Research, London School of Hygiene & Tropical Medicine, Keppel Street, London WC1E 7HT, United Kingdom
3. Department of Infectious Disease Epidemiology, London School of Hygiene & Tropical Medicine, Keppel Street, London, United Kingdom
4. Livingstone Central Hospital, Livingstone, Zambia
5. Department of Infectious Diseases, Veterans Affairs Health Care System, California, USA
6. Department of Parasitology, Leiden University Medical Center, Leiden, The Netherlands
7. Department of Cell and Chemical Biology, Leiden University Medical Center, Leiden, The Netherlands.
8. Longhorn Vaccines and Diagnostics LLC, Bethesda, MD
9. Department of Global Health and Development, London School of Hygiene & Tropical Medicine, Keppel Street, London, United Kingdom
10. Wolfson Wellcome Biomedical Laboratories, Department of Zoology, Natural History Museum, Cromwell Road, London, United Kingdom
11. Department of Gynecology, University of Zambia, Lusaka, Zambia

***Corresponding author:**

Prof. Amaya Bustinduy

London School of Hygiene & Tropical Medicine

Keppel Street

WC1E 7HT- London, UK

Amaya.Bustinduy@lshtm.ac.uk

Word count: 3955

Figures:2

Tables:1

For peer review only - http://bmjopen.bmj.com/site/about/guidelines.xhtml

ABSTRACT

Introduction

Multiplathogen home-based self-sampling offers an opportunity to increase access to screening and treatment in endemic settings with high co-infection prevalence of sexually transmitted (HIV, *Trichomonas vaginalis (Tv),* human papillomavirus [HPV]) and non-sexually transmitted pathogens (*Schistosoma haematobium (Sh)).*Chronic co-infections may lead to disability (female genital schistosomiasis (FGS) and death (cervical cancer). The Zipime-Weka-Schista (Do self-testing sister!) study aims to evaluate the validity, acceptability, uptake, impact and cost-effectiveness of multi-pathogen self-sampling for genital infections among women in Zambia.

Methods and Analysis

This is a longitudinal cohort study aiming to enrol 2,500 non-pregnant, sexually active and non-menstruating women aged 15-50 years from two districts in Zambia with two year follow up. During home visits, community health workers offer HIV and *Tv* self-testing and cervicovaginal self-swabs for (i) HPV by GeneXpert and, (ii) *Sh* DNA detection by conventional (Polymerase Chain Reaction (PCR))and isothermal (Recombinase Polymerase Assay (RPA)) molecular methods. *Schistosoma* ova and circulating anodic antigen are detected in urine. At a clinic follow-up midwives perform the same procedures and obtain hand-held colposcopic images. High-risk -HPV positive women are referred for a two-quadrant cervical biopsy according to age and HIV status. A cost-effectiveness analysis is conducted in parallel.

Ethics and dissemination

The University of Zambia Biomedical Research Ethics Committee (UNZABREC) (reference: 1858-2021), the London School of Hygiene and Tropical Medicine(LSHTM) (reference: 25258) , Ministry of Health and local superintendents approved the study in September 2021 .Written informed consent was obtained from all participants prior to enrolment. Identifiable data collected is stored securely and their confidentiality protected in accordance with the Data Protection Act 1998.

Keywords: Female genital schistosomiasis, *Schistosoma haematobium*, HIV, *Trichomonas vaginalis*, human papillomavirus (HPV), cervical cancer, self-sampling, acceptability, cost-effectiveness, hand-held colposcopy, RPA

For peer review only

Strenghts and Limitations

- The longitudinal design of the study will allow measurements of incidence and persistence of HPV in association with FGS and HIV and its joint contribution to cervical precancer and cancer.
- The one-stop home self-sampling (FGS, HR-HPV) and self-testing (HIV and Trichomonas)approach, if acceptable by participants, can become a scalable integrated screening strategy
- Cost-effectiveness data from the study will inform of community-based integration of FGS within the wider sexual and reproductive health screening in Zambia
- Recruitment may be limited by stigma pertaining to co-existing sexually transmitted infections
- Results may be delayed due to the limited availability of histopathology laboratories

For peer review only

INTRODUCTION

It is estimated that around 45 million women living in sub-Sahara Africa (SSA) are affected by female genital schistosomiasis (FGS), a chronic, neglected and disabling gynaecological disorder associated with infertility, dyspareunia and symptoms mimicking sexually transmitted infections (STIs). (1, 2) FGS is difficult to diagnose and is part of the wider spectrum of urogenital disease caused by the waterborne parasite *Schistosoma haematobium* (*Sh*). In Zambia, reported FGS prevalence ranges from 33-75% of those with urinary schistosomiasis in endemic areas, suggesting a potentially high FGS burden. Data is not yet available on the FGS burden in the general population of Zambia. Furthermore, awareness of the disease and non-sexual mode of transmission is largely absent in endemic communities where information and education are urgently needed. (3)

Conventional FGS diagnosis is challenging as it relies on costly equipment and high-level specialized training is seldom available in resource-limited countries. Accurate estimation of disease burden is therefore hindered. However, the past decade has seen a dramatic increase in knowledge generation on many facets of FGS, and particularly on diagnostic strategies closer to the point-of-care. (4) Studies have shown acceptable sensitivity of genital swabs for the DNA detection of *Sh* from women's genital tracts, potentially offering, for the first time, a scalable diagnostic approach for a parasitic disease that has been unacceptably neglected. (3, 4) Genital self-swabs are well accepted by participants and are known to increase uptake across settings. (5, 6)

There is growing evidence of an association of FGS with prevalent HIV, and suggestion of increased incidence and transmission of HIV in the presence of schistosome infections in women. (7) The lesions and tissue damage caused by FGS can provide an easy route for STI infections. (8) Further, women with schistosome worm infections and with FGS are potentially far more susceptible and at an increased risk of becoming HIV positive. (9) However, there is still a knowledge vacuum on the role of STIs in HIV acquisition in the presence of FGS, despite both STIs and *S.haematobium* infection being shown to be independently associated with HIV acquisition.(8, 10, 11)

For peer review only - http://bmjopen.bmj.com/site/about/guidelines.xhtml

For peer review only

There have been small studies suggesting an increased prevalence of high risk (HR-) HPV, the etiological agent of cervical cancer, in women with FGS compared to those without ,(12) and higher odds of having an abnormal visual examination with acetic acid (VIA)(13) and some suggestion of potential links between FGS and cervical cancer. (14, 15) In Zambia, cervical cancer is the most common female cancer, particularly in women aged 15 to 44 years(16, 17) and with high associated mortality (43.4 per 100,000 women). The lack of accessible screening and treatment programs, (18) and HPV vaccination contribute to the high cervical cancer incidence in these settings. (19) HPV-DNA based tests are recommended for primary screening of cervical cancer as part of the WHO 2021 cervical cancer screening and treatment guidelines (20), as better strategies than visual diagnosis. (21, 22) Despite validated self-sampling strategies for the detection of oncogenic or HR-HPV genotypes, community-based screening is not yet widely adopted alone or integrated with screening for other pathogens due to the high cost and infrastructure requirements of currently available tests. (23)

This study protocol describes the development and costing of a community-based comprehensive package for SRH screening in Zambia, including sexually (HPV, HIV, *Tv*) and non-sexually transmitted infections (*Sh)* in different endemicity settings with the long-term aim of preventing life threatening and disabling conditions such as cervical cancer, HIV and FGS. The overall justification of this study lies in the dearth of research exploring the feasibility of integrated screening and testing strategies of genital co-infections, and the epidemiological associations between them.(24-26)

METHODS AND DESIGN

Study design

The Zipime Weka Schista (Do self-testing sister!) study is a longitudinal cohort designed as a holistic approach to FGS detection at scale in the community. The study design will test the hypothesis that integration of home self-sampling for the screening of FGS in conjunction with HR-HPV and self-testing for HIV and STIs is an diagnostically accurate, cost-effective and self-empowering strategy that will increase the detection of cases and improve access to care for women of reproductive age in SSA.

The two main aims of the Zipime Weka Schista study are:

For peer review only - http://bmjopen.bmj.com/site/about/guidelines.xhtml

1. To assess the performance of genital self-sampling, compared to healthcare provider sampling obtained for the detection of *Sh* by molecular testing (real-time PCR and Recombinase Polymerase Assay (RPA)) across two different *Sh* endemicity settings in Zambia, and
2. To assess the observed costs of integrating community based multiple pathogen genital self-sampling (*Sh*, HPV, STIs) and oral self-test (HIV) into one-stop home intervention and. project costs at scale.

Our secondary aims are to explore the epidemiological and immunological interrelationships between FGS and HPV as a potential risk factor for cervical cancer and expand knowledge on HIV acquisition in the presence of co- infections. We will also measure acceptability and feasibility of home-based multiple genital self-sampling and to measure HIV linkage to care and cervical cancer management after home self-sampling and testing. We will further aim to evaluate the effect of FGS and other STI on the diagnostic accuracy of visual inspection for detection of cervical precancer (CIN2+, CIN3+) and to describe the association of FGS, HPV, HIV and STIs with the presence concentration, diversity, and relative abundance of other key cervicovaginal microbiota and infections. We will also aim to consolidate testing for FGS and HPV into one field-applicable multiplex molecular assay and to recruit a sub-set of male household members of recruited participants screening male genital schistosomiasis.

Study setting and population

The study is being conducted in Lusaka and Southern provinces of Zambia. The specific study study areas are two sites in Kafue district with high (estimated *Sh* prevalence >70%) and moderate (estimated *Sh* prevalence >50%) prevalence and another site in Livingstone district with low prevalence (estimated *Sh* prevalence <10%). Each woman will have one home visit and one clinic visit at baseline with annual follow up for 2 years. Enrolment of eligible women began in January 2022 and is expected to be completed in May of 2024.

Inclusion/Exclusion criteria

All non-pregnant, sexually active women aged 15-50 years who are currently not menstruating at the time of recruitment and sampling and are able to provide consent are invited to take part in the study. In addition, women should be Zambian residents of the endemic study area for at least one year.Women are

For peer review only - http://bmjopen.bmj.com/site/about/guidelines.xhtml
For peer review only

excluded prior to their sexual debut ,pregnant or currently menstruating at the time of recruitment and sampling.

Sampling strategy and data collection procedures

Community-based cluster sampling was used to obtain a random sample of participants from the study communities. Communities and their households were mapped and each community was subdivided into blocks of approximately 50 households; each one would yield approximately 30 enrolled women. These community blocks were the primary sampling units, with all households in a selected block visited, and all eligible women aged 15-50 years invited to take part. Blocks were randomly selected using random number generation in Stata, and are being visited sequentially in the order that they were randomly selected. Participating women are assigned a unique identifier. Field data are entered using the London School of Hygiene and Tropical Medicine (LSHTM) ODK system and uploaded to a secure server in Zambia. The data are then transferred to a LSHTM secure server. The Chief Investigator is responsible for the data analysis with the support of the Department of Infectious Diseases Epidemiology at LSHTM.

Sample size and study power

A total of 2,500 women will be randomly selected from two districts (1,250 from each district). Based on previous assessments of FGS using PCR testing of genital samples from women in the study setting, we assume that 5-10% of genital samples from healthcare provider sampling will have *Sh* detected by molecular testing (5% in the lower endemicity area, 10% in the higher endemicity area). Taking clinic-based sampling as the gold standard, allowing for 10% loss to follow-up between the home and clinic sampling, and assuming that the diagnostic sensitivity and specificity of self-sampling compared to clinic-based sampling for the detection of *Sh* are 70% and 85%, respectively, we will be able to estimate the assumed target diagnostic sensitivity of 70% with a 95% CI of 62.3-76.6% overall, and the assumed target diagnostic specificity of 85% with a 95% CI of 83.4-86.5% (using the Clopper-Pearson formula to calculate an exact 95% confidence interval for this proportion). Considering individual districts, we will be able to estimate sensitivity of 70% with a 95% CI of 60.6-78.2% in the higher endemicity area and a 95% CI of 55.9-81.2%

For peer review only - http://bmjopen.bmj.com/site/about/guidelines.xhtml

in the lower endemicity area. Corresponding confidence intervals (CI) for specificity would be 83-87% in each area.

FIELD PROCEDURES

Community sensitization activities take place to inform target communities of the study aims and promote voluntary participation. Different support materials are used. Some of these include local drama groups, information brochures and an FGS manual edited by Zambart. Study community workers (SCWs) register expressions of interest and conduct home visits within the randomized zones to women that have expressed interest in participating in the study. All SCWs are females. Every participant has a total of three visits during the study: baseline, 12, and 24 months from recruitment. A schematic study flow is shown in **Figure 1**.

<< Insert Figure 1 here>>

Home visits

Home visits are conducted by SCWs who perform a demonstration of self-sampling, using a 3D model for illustration (supplementary figure 1). SCWs also obtain consent from participants and administer demographic and exposure questionnaires. After obtaining informed consent and completing questionnaires on demography, clinical symptoms and other relevant health and water contact information, the SCWs offer partcipants two genital self-swabs. The first swab is stored in PrimeStore® MTM molecular transport media (donated by Longhorn Vaccines and Diagnostics LLC, Bethesda, MD) for for the detection of *Sh* by molecular methods (PCR and RPA). The second swab is stored in Cytopreserve® for HR-HPV detection by Xpert (27) Participants are also offered two commercial self-tests: one oral (saliva) for HIV (28) and one vaginal self-swab for *Trichomonas vaginalis* (*Tv*). (29) For both self-tests, results are read by the SCW and given immediately to the participant. After all the self-sampling swabs are delivered, the SCW record acceptability results based on a Likert Scale. A confirmatory test is performed on site if there is a positive self-test for HIV. All HIV-positive women are referred to the local clinic for further management and linkage to care. For *Tv*, women are referred for treatment at the clinic visit. A urine sample is analysed via microscopy for *Sh* ova detection and circulating anodic antigen (CAA).(30)

For peer review only - http://bmjopen.bmj.com/site/about/guidelines.xhtml

For peer review only

Clinic visit

A follow up visit takes place at a local clinic. A trained healthcare provider (midwife) collects two cervicovaginal swabs and 5 ml of cervicovaginal lavage (CVL). Swabs are placed in PrimeStore® MTM molecular transport media (donated by Longhorn Vaccines and Diagnostics LLC, Bethesda, MD) and CVL are stored at -20 C on site for future detection of : 1) *Sh* DNA by real-time PCR as the reference assay for FGS; (31); 2) *Sh* DNA by Xpert® CT/NG (Chlamydia Trachomatis/Neisseria gonorrhoeae) (32) and studies of biomarkers and cervivovaginal microbiome. Approximately 5 ml of blood is collected by venipuncture in an EDTA tube for the detection of *Schistosoma* and STI serology. A hand-held colposcope, with an enhanced visual assessment (EVA) system is used to image the participant's cervix and vagina (MobileODT, Tel Aviv, Israel).Praziquantel and metronidazole are available free of charge for study partcipants with any positive result.(33)

Biopsy procedure

Women testing positive for any high-risk HPV by PCR (Xpert® HPV) during the home visit and depending on their age and HIV status (see below) are referred for colposcopy. Visual inspection using acetic acid and lugol's iodine (VIA/VILI) is performed by a trained gynecologist before biopsy of visible lesions. If no lesions are visible, two random biopsies are taken from normal areas including the transformation zone (at 12 and 6 o'clock), **Figure 2.** Cervical biopsies will be processed at the pathology laboratory at National Health laboratory Service, Johannesburg, South Africa and read using the 2-tier CIN classification system.

<< Insert Figure 2 here>>

HIV-negative women under the age of 25 and women living with HIV (WLHIV) under the age of 20 years have all procedures performed, including HPV testing in the community, but they do not undergo biopsy in clinic despite positive HR-HPV results. (34) This strategy follows ethical concerns of unnecessary biopsy and management of cervical lesions based on known HPV clearance and low risk of cervical precancer in young women. (35) For these women, there is annual longitudinal follow up for two years with repeated infection and clinical re-assessments.

For peer review only - http://bmjopen.bmj.com/site/about/guidelines.xhtml

***Women diagnosed with cervical precancer and invasive cervical cancer:*.** If found to have histopathology verified CIN2+, participants are recalled for CIN2+ management. This visit is scheduled at the earliest convenient date once the result is known . In Zambia, all cancer patients are referred to the Cancer Diseases Hospital (CDH) based in Lusaka. Patients are filtered through the district and provincial hospitals serving as primary centres for cervical cancer screening countrywide before being referred to CDH for definitive treatment. Treatment at CDH is done according to the nationally and globally developed guidelines.(36)

LABORATORY PROCEDURES

The collection, storage and processing of all study samples is summarised in **Table 1**. Urine samples are processed for; 1) ***Urine parasitology*** Approximately 10mL (minimum) of urine is processed by centrifugation to detect *Sh* eggs by microscopy performed at the local government clinics in Kafue and Livingstone in Zambia by a trained technician and 2). ***CAA antigen assay.*** 2 mL of urine are stored at 4 ^{o}C and shipped to Lusaka and further to Leiden University Medical Center, The Netherlands. (37)

All genital samples (home/clinic) are processed for; 1) ***Detection of Schistosoma DNA*** using ITS-2 Real-time PCR processed at Zambart laboratories in Lusaka, Zambia.(31, 38) From the DNA already extracted for PCR additional well-validated qPCR assays will be used (39) to characterize the vaginal microbiota by quantifying key species and 2) ***Detection of Schistosoma DNA* using *RPA***. Up to 2 mL of fluid from vaginal lavage is stored at -20^{0}C and shipped to Lusaka. This assay is processed at Zambart laboratory in Zambia (40, 41) and 3) ***Inflammatory Markers***. Up to 3 mL of fluid from vaginal lavage is stored at -80 ^{o}C and shipped to Lusaka. (25, 31, 42)

<< Insert Table 1 here>>

Development of a multiplex assay for *S. haematobium* and HR-HPV testing

In a exploratory study we will aim to integrate into one molecular assay the RPA for *Sh* and HPV to increase accessibility and feasibility of molecular dual pathogen screenings after encouraging results on the validaty of the CVL RPA assay against standard real-time PCR,(43) which could be coupled with oncogenic HPV genotypes. (44)

For peer review only

For peer review only - http://bmjopen.bmj.com/site/about/guidelines.xhtml

Qualitative assessment

In-Depth feasibility and acceptability of integrated sampling, testing and treatment

Previous work has documented the acceptability of performing multiple genital swabs at a single encounter .(27) For the current proposal, in-depth cognitive interviews will be delivered to a subset of participants to evaluate the acceptability of the multi-pathogen self-swabbing strategy (three genital swabs and one oral swab). (45)

STATISTICAL ANALYSIS

To assess the performance of genital self-sampling compared to healthcare provider sampling for the detection of *Sh,* sensitivity, specificity, PPV and NPV will be calculated together with 95% CIs. Results will be presented overall and stratified by geographic setting. To explore associations of baseline FGS with prevalence of cervical precancer at baseline and cervical precancer incidence at 12 and 24 months, logistic regression will be used to estimate odds ratios. Associations between baseline FGS and HR-HPV persistence at 12 months will be estimated with generalized estimating equation to account for multiple HR-HPV infection and multiple infection states (persistence and clearance).(46) The association of FGS with HIV acquisition will be assessed using Poisson regression, controlling for key confounders.

Other outcome measures obtained in the study will include 1)HIV incidence rate, HR-HPV clearance and persistence, prevalence and incidence of cervical precancer (histology confirmed CIN2 or CIN3+), measures of association between different exposures (reproductive tract infections); 2) Acceptability of multiple genital self-sampling interventions ; 3) Rates of antiretroviral (ART) initiation, continuation of treatment and access to care ; 4) Cervical precancer diagnosis and treatment, testing and management of STIs. 4) Presence or absence of cervicovaginal microbiota in women with FGS, HPV and STIs. Expressed as relative abundance (percentage abundance of each species), genomic concentration per microorganism (genome equivalents per mL); 5) Sensitivity, specificity, PPV, NPV of visual inspection of the cervix according to FGS status and Integrated molecular field-applicable testing platform for *Sh* and HR-HPV.

For peer review only - http://bmjopen.bmj.com/site/about/guidelines.xhtml

Quality control

Several procedures have been employed to ensure the quality of the study data, maximizing the validity and reliability of the data obtained and of the evaluation of the results: These include the following (a) 10 % of all samples (positive and negative results from diagnostic assays) will be reviewed blindly by experts at different institutions; parasitology (Liverpool School of Tropical Medicine, Liverpool, UK), PCR (Natural History Museum, London, UK), colposcopic images (University of Zambia, Lusaka, Zambia).

Economic analysis

A full economic costing from a provider's and patient's perspective is ongoing alongside data collection. Cost data is collected combining a bottom-up and top-down approach following established costing guidelines, (47) and STI self-testing economic evaluations in the.region. This allows us to estimate the total intervention cost, and unit costs per: woman screened, FGS infection detected, per case of CIN2+ detected, per person linked to care, per person successfully treated overall and per pathogen identified. We will model the costs across a set of scale-up scenarios to understand the budget implications of introducing widescale self-screening. Populating a health economics decision model with the study outcomes, we will identify cost thresholds under which joint self-sampling for FGS, HPV, STIs and HIV is cost-effective. This will be relevant for informing the maximum threshold diagnostic kit price based on its potential cost-effectiveness.(48) MoH and policy makers will be informed on the most cost-effective screening strategy to implement for FGS screening. This study constitutes the first economic evaluation of FGS.

Patient and Public involvement

Participants who are being recruited to this study did not take part in the design or implementation of the study. They are however deeply involved finding solutions for better follow up through focus groups discussions and mobilisation activities in the community.

DISCUSSION

For peer review only

Sexual and reproductive health is a critical area of development highlighted in the United Nations Sustainable Development Goal 3. There is an estimated 40 million women unknowingly living with FGS, with dire consequences for their sexual and reproductive lives which include infertility, pain with sexual relations, ectopic pregnancies and increased risk of HIV acquisition and cervical precancer development, mostly in countries with high prevalence of STIs. This study will provide high quality evidence on the validity, acceptability, uptake and costs of integrating community based multiple-pathogen genital self-sampling (Sh, HR-HPV, STIs) and oral self-test (HIV) into one-stop intervention. Findings will help inform policy and practice at local and regional levels. Importantly, this study brings together existing and new partnerships for a multidisciplinary investigative approach, to explore the value of integration of reproductive health screening practices through self-sampling and testing. The baseline results will provide further validation of self-swabs as an acceptable sampling strategy for FGS diagnosis in a larger sample population and with a wider scope than previously performed. Its longitudinal approach will also provide an allied impact assessment on the incidence of cervical precancer, HIV and STIs and early HIV detection and access to treatment for the wider improvement of women's SRH in Zambia. This is important within the growing evidence of a link between FGS and cervical cancer. (15) The clear interplay between genital infections highlights the urgent need to bolster programmes that jointly integrate strategies to screen and detect multiple genital infections with simple, affordable and as close to the point-of-care as possible.

Genital self-sampling and self-testing through community based programmes are widely accepted strategies for the detection of devastating diseases like HIV in both adults and adolescents. (8, 31, 49) HIV self-testing programmes have been shown to be cost-effective. Genital self-sampling has enhanced access to health services among hard-to-reach populations such as young people, (31) and those who do not regularly access health screening services. (1, 50) It also empowers women and protect their privacy since self-swabs can be performed at home or in other private places and increases compliance with testing. A high proportion of women, including those from resource-limited settings have been found to prefer vaginal specimen self-collection (51) compared with clinic-based sampling. In addition to acceptability, two other factors make genital self-sampling advantageous; 1) the availability of vaginal self-sampling is

For peer review only - http://bmjopen.bmj.com/site/about/guidelines.xhtml

effective for improving participation in specific SRH screening programmes and 2) the sensitivity of PCR-based assays on self-collected specimens compares favourably with physician-performed sampling.(1)

The synergistic approach proposed in this study is justified by the known and suspected interactions of the different genital co-infections. FGS is associated with genital inflammation, friable blood vessels and contact bleeding. (52-54) The disruptions of the mucosal surface in FGS may place women at risk for HIV, as has been found for several STIs. (55-57) Women with urogenital schistosomiasis had a 3-4-fold higher odds of prevalent HIV in cross-sectional studies in Zimbabwe, Tanzania and Mozambique. (58-62) However, FGS as a risk factor for HIV is not yet integrated in ongoing screening programmes, including HIV self-testing strategies. (63-65)

Current FGS diagnostic strategies are limited because they require expertise and equipment that may not be readily available in low income settings. Several diagnostics will be tested in this study to provide 'real-world' data on the scalability of the different approaches. Hand-held colposcopy as a low-cost device has been used in both cervical cancer screening and FGS studies. (4, 66) However, expert review is still required for the identification of characteristic FGS lesions and inter-rater correlation is poor. (67) This study will enhance diagnostics by applying multipathogen DNA testing with conventional molecular assays that are field deployable, such as the RPA (32, 40) Following on the positive correlation between RPA assay from CVL against standard real-time PCR, (68) in the Schista study we will aim to develop a multiplex RPA assay for Sh and HR-HPV to increase accessibility and feasibility of dual pathogen molecular screenings.

To make interventions ultimately sustainable these need to be cost-effective. In this study we aim to assess for the first time the observed costs of integrating community based multiple-pathogen genital self-sampling (Sh, HPV, STIs) and oral self-test (HIV) in a one-stop intervention and project costs at scale for the justification of the diagnostic kit price based on its impact on disease prevalence. (26)

To conclude, the Zipime Weka Schista study has a number of potential benefits at both individual and community levels including diagnosis and treatment of previously undiagnosed S. haematobium infection as well as diagnosis, treatment and linkage to care for cervical cancer, HIV and STIs. Multi-pathogen self-

For peer review only - http://bmjopen.bmj.com/site/about/guidelines.xhtml

sampling and testing constitutes an unmissable opportunity to offer potentially life-changing health services closer to the users.(6)

.

DECLARATIONS

Competing interest statement

The authors declare they have no competing interests.

Data availability statement

Data will be available upon request on LSHTM Data Compass

Funding statement

This study is funded through a UKRI Future Leaders Fellowship (MR/T041900/1) awarded to Prof. AL Bustinduy. Prof E Webb and Prof R Hayes received funding from MRC Grant Reference MR/K012126/1. This award is jointly funded by the UK Medical Research Council (MRC) and the UK Department for International Development (DFID) under the MRC/DFID Concordat agreement and is also part of the EDCTP2 programmme supported by the European Union. Funders had no role in study design, data collection and analysis, decision to publish, or preparation of the manuscript.

Authors' Contributions

AB originally conceived and designed the study in consultation with PM, HA, BW, IH, FTP and RH. KS wrote the first draft of this manuscript. BW, HK and AS advised on primary and some secondary objectives, FTP and OL conceived the economic analysis, EW and NK helped design the statistical framework of the study and contributed to all drafts of the manuscript. BK, BW, LVL, JF, GVD and PC contributed to the laboratory component of the study. RN VB and BVcontributed to the design of secondary objectives. All authors read and approved the final manuscript.

Acknowledgements

We would like to thank and acknowledge all the participants, Schista! study fieldworkers and midwives. and the wider Schista! study team who contributed towards the design of the study. Also, our partners in Zambia including the Ministry of Health and District Health Management Teams; the administrative and support teams at Zambart and LSHTM.

REFERENCES

For peer review only

1. Kjetland EF, Kurewa EN, Mduluza T, et al. The first community-based report on the effect of genital Schistosoma haematobium infection on female fertility. *Fertil Steril*. 2010;94:1551-1553.
2. Bustinduy AL, Randriansolo B, Sturt AS, et al. Chapter One - An update on female and male genital schistosomiasis and a call to integrate efforts to escalate diagnosis, treatment and awareness in endemic and non-endemic settings: The time is now. In: Rollinson D, Stothard R, eds. *Advances in Parasitology*: Academic Press; 2022:1-44.
3. Hotez PJ, Harrison W, Fenwick A, et al. Female genital schistosomiasis and HIV/AIDS: Reversing the neglect of girls and women. *PLoS Negl Trop Dis*. 2019;13:e0007025.
4. Sturt AS, Webb EL, Phiri CR, et al. Genital self-sampling compared with cervicovaginal lavage for the diagnosis of female genital schistosomiasis in Zambian women: The BILHIV study. *PLoS Negl Trop Dis*. 2020;14:e0008337.
5. Mbatha JN, Galappaththi-Arachchige HN, Mtshali A, et al. Self-sampling for human papillomavirus testing among rural young women of KwaZulu-Natal, South Africa. *BMC Res Notes*. 2017;10:702.
6. Francis SC, Mthiyane TN, Baisley K, et al. Prevalence of sexually transmitted infections among young people in South Africa: A nested survey in a health and demographic surveillance site. *PLoS Med*. 2018;15:e1002512.
7. Wall KM, Kilembe W, Vwalika B, et al. Schistosomiasis is associated with incident HIV transmission and death in Zambia. *PLoS Negl Trop Dis*. 2018;12:e0006902.
8. Kjetland EF, Ndhlovu PD, Gomo E, et al. Association between genital schistosomiasis and HIV in rural Zimbabwean women. *AIDS*. 2006;20:593-600.
9. Downs JA, Dupnik KM, van Dam GJ, et al. Effects of schistosomiasis on susceptibility to HIV-1 infection and HIV-1 viral load at HIV-1 seroconversion: A nested case-control study. *PLoS Negl Trop Dis*. 2017;11:e0005968.
10. Downs JA, Mguta C, Kaatano GM, et al. Urogenital schistosomiasis in women of reproductive age in Tanzania's Lake Victoria region. *Am J Trop Med Hyg*. 2011;84:364-369.
11. Wall KM, Kilembe W, Vwalika B, et al. Schistosomiasis is associated with incident HIV transmission and death in Zambia. *PLOS Neglected Tropical Diseases*. 2018;12:e0006902.
12. Kjetland EF, Ndhlovu PD, Mduluza T, et al. The effects of genital schistosoma haematobium on human papillomavirus and the development of cervical neoplasia after five years in a Zimbabwean population. *Eur J Gynaecol Oncol*. 2010;31:169-173.
13. Rafferty H, Sturt AS, Phiri CR, et al. Association between cervical dysplasia and female genital schistosomiasis diagnosed by genital PCR in Zambian women. *BMC Infect Dis*. 2021;21:691.
14. Vlassoff C, Arogundade K, Patel K, Jacobson J, Gyapong M, Krentel A. Improving the Response of Health Systems to Female Genital Schistosomiasis in Endemic Countries through a Gender-Sensitive Human Rights-Based Framework. *Diseases*. 2022;10:125.
15. Engels D, Hotez PJ, Ducker C, et al. Integration of prevention and control measures for female genital schistosomiasis, HIV and cervical cancer. *Bull World Health Organ*. 2020;98:615-624.
16. Bray F, Ferlay J, Soerjomataram I, Siegel RL, Torre LA, Jemal A. Global cancer statistics 2018: GLOBOCAN estimates of incidence and mortality worldwide for 36 cancers in 185 countries. *CA Cancer J Clin*. 2018;68:394-424.
17. Bruni L AG, Serrano B, Mena M, Gómez D, Muñoz J, Bosch FX, de Sanjosé S. . Human Papillomavirus and Related Diseases in Zambia. Summary Report 17 June 2019. 2019. Accessed 15th October, 2019.
18. Bruni L, Serrano B, Roura E, et al. Cervical cancer screening programmes and age-specific coverage estimates for 202 countries and territories worldwide: a review and synthetic analysis. *Lancet Glob Health*. 2022;10:e1115-e1127.
19. Bruni L, Saura-Lázaro A, Montoliu A, et al. HPV vaccination introduction worldwide and WHO and UNICEF estimates of national HPV immunization coverage 2010-2019. *Prev Med*. 2021;144:106399.
20. World Health Organization (2021). WHO guideline for screening and treatment of cervical pre-cancer lesions for cervical cancer prevention, 2nd ed. World Health Organization. https://apps.who.int/iris/handle/10665/342365. License: CC BY-NC-SA 3.0 IGO.
21. Arbyn M, Sankaranarayanan R, Muwonge R, et al. Pooled analysis of the accuracy of five cervical cancer screening tests assessed in eleven studies in Africa and India. *Int J Cancer*. 2008;123:153-160.

For peer review only

22. Arbyn M, Simon M, Peeters E, et al. 2020 list of human papillomavirus assays suitable for primary cervical cancer screening. *Clin Microbiol Infect*. 2021;27:1083-1095.
23. Arbyn M, Smith SB, Temin S, et al. Detecting cervical precancer and reaching underscreened women by using HPV testing on self samples: updated meta-analyses. *BMJ*. 2018;363:k4823.
24. Mezei AK, Pedersen HN, Sy S, et al. Community-based HPV self-collection versus visual inspection with acetic acid in Uganda: a cost-effectiveness analysis of the ASPIRE trial. *BMJ Open*. 2018;8:e020484.
25. Lunny C, Taylor D, Hoang L, et al. Self-Collected versus Clinician-Collected Sampling for Chlamydia and Gonorrhea Screening: A Systemic Review and Meta-Analysis. *PLoS One*. 2015;10:e0132776.
26. Mangenah C, Mwenge L, Sande L, et al. Economic cost analysis of door-to-door community-based distribution of HIV self-test kits in Malawi, Zambia and Zimbabwe. *J Int AIDS Soc*. 2019;22 Suppl 1:e25255.
27. Rutty Phiri C, Sturt AS, Webb EL, et al. Acceptability and feasibility of genital self-sampling for the diagnosis of female genital schistosomiasis: a cross-sectional study in Zambia. *Wellcome Open Res*. 2020;5:61.
28. Zachary D, Mwenge L, Muyoyeta M, et al. Field comparison of OraQuick ADVANCE Rapid HIV-1/2 antibody test and two blood-based rapid HIV antibody tests in Zambia. *BMC Infect Dis*. 2012;12:183.
29. Campbell L, Woods V, Lloyd T, Elsayed S, Church DL. Evaluation of the OSOM Trichomonas rapid test versus wet preparation examination for detection of Trichomonas vaginalis vaginitis in specimens from women with a low prevalence of infection. *J Clin Microbiol*. 2008;46:3467-3469.
30. van Dam GJ, de Dood CJ, Lewis M, et al. A robust dry reagent lateral flow assay for diagnosis of active schistosomiasis by detection of Schistosoma circulating anodic antigen. *Exp Parasitol*. 2013;135:274-282.
31. Kjetland EF, Hove RJ, Gomo E, et al. Schistosomiasis PCR in vaginal lavage as an indicator of genital Schistosoma haematobium infection in rural Zimbabwean women. *Am J Trop Med Hyg*. 2009;81:1050-1055.
32. Garrett N, Mitchev N, Osman F, et al. Diagnostic accuracy of the Xpert CT/NG and OSOM Trichomonas Rapid assays for point-of-care STI testing among young women in South Africa: a cross-sectional study. *BMJ Open*. 2019;9:e026888.
33. Ministry of Health. National cancer control strategic plan 2016-2021. Lusaka: Zambia 2017. Available at: https://www.iccp-portal.org/system/files/plans/NCCSP%20Final%20Version%20Zambia.pdf,. Accessed 5th October, 2022.
34. Parham GP, Mwanahamuntu MH, Kapambwe S, et al. Population-level scale-up of cervical cancer prevention services in a low-resource setting: development, implementation, and evaluation of the cervical cancer prevention program in Zambia. *PLoS One*. 2015;10:e0122169.
35. Woodman CB, Collins S, Winter H, et al. Natural history of cervical human papillomavirus infection in young women: a longitudinal cohort study. *Lancet*. 2001;357:1831-1836.
36. Mumba JM, Kasonka L, Owiti OB, et al. Cervical cancer diagnosis and treatment delays in the developing world: Evidence from a hospital-based study in Zambia. *Gynecol Oncol Rep*. 2021;37:100784.
37. Corstjens PL, van Lieshout L, Zuiderwijk M, et al. Up-converting phosphor technology-based lateral flow assay for detection of Schistosoma circulating anodic antigen in serum. *J Clin Microbiol*. 2008;46:171-176.
38. Obeng BB, Aryeetey YA, de Dood CJ, et al. Application of a circulating-cathodic-antigen (CCA) strip test and real-time PCR, in comparison with microscopy, for the detection of Schistosoma haematobium in urine samples from Ghana. *Ann Trop Med Parasitol*. 2008;102:625-633.
39. Jespers V, van de Wijgert J, Cools P, et al. The significance of Lactobacillus crispatus and L. vaginalis for vaginal health and the negative effect of recent sex: a cross-sectional descriptive study across groups of African women. *BMC Infect Dis*. 2015;15:115.
40. Rosser A, Rollinson D, Forrest M, Webster BL. Isothermal Recombinase Polymerase amplification (RPA) of Schistosoma haematobium DNA and oligochromatographic lateral flow detection. *Parasit Vectors*. 2015;8:446.

For peer review only - http://bmjopen.bmj.com/site/about/guidelines.xhtml

41. Utzinger J, Becker SL, van Lieshout L, van Dam GJ, Knopp S. New diagnostic tools in schistosomiasis. *Clin Microbiol Infect*. 2015;21:529-542.
42. Sturt AS, Webb EL, Patterson C, et al. Cervicovaginal Immune Activation in Zambian Women With Female Genital Schistosomiasis. *Front Immunol*. 2021;12:620657.
43. Archer J, Patway FK, Sturt AS, et al. Validation of the isothermal Schistosoma haematobium Recombinase Polymerase Amplification (RPA) assay, coupled with simplified sample preparation, for diagnosing female genital schistosomiasis using cervicovaginal lavage and vaginal self-swab samples. *PLOS Neglected Tropical Diseases*. 2022;16:e0010276.
44. Ma B, Fang J, Wang Y, et al. Isothermal Method of a Recombinase Polymerase Amplification Assay for the Detection of Most Common High-Risk Human Papillomavirus Type 16 and Type 18 DNA. *Clin Lab*. 2017;63:27-38.
45. Simwinga M KM, Dacombe RJ, Kayira L, Muzumara A, Johnson CC, et al. Ability to understand and correctly follow HIV self-test kit instructions for use: applying the cognitive interview technique in Malawi and Zambia. *J Int AIDS Soc*.22 Suppl 1:e25253.
46. Xue X, Gange SJ, Zhong Y, et al. Marginal and Mixed-Effects Models in the Analysis of Human Papillomavirus Natural History Data. *Cancer Epidemiology, Biomarkers & Prevention*. 2010;19:159 - 169.
47. Vassall A, Sweeney, S., Kahn, J., Gomez, G. B., Bollinger, L., Marseille, E., … & Levin, C, . Reference Case for Estimating the Costs of Global Health Services and Interventions. Global Health Cost Consortium. 2017. Available at: https://ghcosting.org/pages/standards/reference_case Accessed 15th March, 2022.
48. Terris-Prestholt F, Foss AM, Cox AP, et al. Cost-effectiveness of tenofovir gel in urban South Africa: model projections of HIV impact and threshold product prices. *BMC Infect Dis*. 2014;14:14.
49. Kjetland EF, Poggensee G, Helling-Giese G, et al. Female genital schistosomiasis due to Schistosoma haematobium. Clinical and parasitological findings in women in rural Malawi. *Acta Trop*. 1996;62:239-255.
50. Pillay P, Downs JA, Changalucha JM, et al. Detection of Schistosoma DNA in genital specimens and urine: A comparison between five female African study populations originating from S. haematobium and/or S. mansoni endemic areas. *Acta Tropica*. 2020;204:105363.
51. Helling-Giese G, Kjetland EF, Gundersen SG, et al. Schistosomiasis in women: manifestations in the upper reproductive tract. *Acta Tropica*. 1996;62:225-238.
52. Yeh HY, Mitchell PD. Ancient Human Parasites in Ethnic Chinese Populations. *The Korean journal of parasitology*. 2016;54:565-572.
53. Barakat RM. Epidemiology of Schistosomiasis in Egypt: Travel through Time: Review. *Journal of advanced research*. 2013;4:425-432.
54. Madden F. A case of Bilharzia of the vagina. *Lancet*. 1899;1:1716-1710.
55. Kaul R, Pettengell C, Sheth PM, et al. The genital tract immune milieu: an important determinant of HIV susceptibility and secondary transmission. *J Reprod Immunol*. 2008;77:32-40.
56. Masson L, Passmore JA, Liebenberg LJ, et al. Genital inflammation and the risk of HIV acquisition in women. *Clin Infect Dis*. 2015;61:260-269.
57. Kjetland EF, Hegertun IE, Baay MF, Onsrud M, Ndhlovu PD, Taylor M. Genital schistosomiasis and its unacknowledged role on HIV transmission in the STD intervention studies. *International journal of STD & AIDS*. 2014;25:705-715.
58. Kjetland EF, Leutscher PD, Ndhlovu PD. A review of female genital schistosomiasis. *Trends Parasitol*. 2012;28:58-65.
59. Downs JA, Mguta C, Kaatano GM, et al. Urogenital schistosomiasis in women of reproductive age in Tanzania's Lake Victoria region. *Am J Trop Med Hyg*. 2011;84:364-369.
60. Hotez PJ, Fenwick A, Kjetland EF. Africa's 32 cents solution for HIV/AIDS. *PLoS Negl Trop Dis*. 2009;3:e430.
61. Brodish PH, Singh K. Association between *Schistosoma haematobium* exposure and Human Immunodeficiency Virus infection among females in Mozambique. *Am J Trop Med Hyg*. 2016;94:1040-1044.

For peer review only - http://bmjopen.bmj.com/site/about/guidelines.xhtml

62. Ndeffo Mbah ML, Poolman EM, Drain PK, Coffee MP, van de Werf MJ, Galvani AP. HIV and Schistosoma haematobium prevalences correlate in sub-Saharan Africa. *Tropical Medicine and International Health*. 2013.
63. Berge ST, Kabatereine NB, Gundersen SG, et al. Generic praziquantel in South Africa: The necessity for policy change to avail cheap, safe and efficacious schistosomiasis drugs to the poor, rural population *Southern Afr J Epidemiol Infect*. 2011;26:22-25.
64. Lothe A. Treating Bilharzia among High School Pupils. A study of opportunities and constraints for treating Bilharzia among high school pupils in Ugu district, South Africa. *Faculty of Economics and Social Sciences*. Development Management: Agder University College; 2012:112.
65. Ndhlovu PD, Mduluza T, Kjetland EF, et al. Prevalence of urinary schistosomiasis and HIV in females living in a rural community of Zimbabwe: does age matter? *Transactions of the Royal Society of Tropical Medicine and Hygiene*. 2007;101:433-438.
66. Taghavi K, Moono M, Mwanahamuntu M, et al. Screening test accuracy to improve detection of precancerous lesions of the cervix in women living with HIV: a study protocol. *BMJ Open*. 2020;10:e037955.
67. Sturt A, Bristowe H, Webb E, et al. Visual diagnosis of female genital schistosomiasis in Zambian women from hand-held colposcopy: agreement of expert image review. *Wellcome Open Res*. 2023;8:14.
68. Archer J, Patwary FK, Sturt AS, et al. Validation of the isothermal Schistosoma haematobium Recombinase Polymerase Amplification (RPA) assay, coupled with simplified sample preparation, for diagnosing female genital schistosomiasis using cervicovaginal lavage and vaginal self-swab samples. *PLoS Negl Trop Dis*. 2022;16:e0010276.

Figure legends

Figure 1. Schista study flow

Figure 2. Flowchart for cervical precancer determination in HIV negative an women living with HIV

Supplementary Information

Supplemental figure:3D models for the teaching of self sa

For peer review only

Table 1: Sample storage and processing for the Schista study.

*High risk HPV by self-collected swab only

Sample	Location	Storage	Sample Processing	Location of processing
**Self-collected swab Provider-collected swab**	Home	Room temperature (Stored in Primestore media®)	Sh DNA (PCR, RPA)<br><br>GeneXpert- High Risk HPV DNA*	Zambart (Zambia)
**Urine for microscopy**	Home	Room temperature	Sh eggs<br>Urine haemastix	District Laboratory (Kafue, Livingstone, Zambia)
**Urine stored for CAA**	Clinic	4 °C	Circulating Anodic Antigen	Leiden University Medical Centre (The Netherlands)
**Cervicovaginal lavage**	Clinic	-20 °C	Sh DNA (PCR, RPA)<br><br>GeneXpert-CT/NG<br><br>Microbiota	Zambart (Zambia)
**Blood for serology**	Clinic	-20 °C	Sh, STI Antibody	Zambart (Zambia)
**Colposcopy images**	Clinic	Encrypted server	Expert Review<br>Computer Analysis	Zambia<br>London School of Hygien & Tropical Medicine (UK)

For peer review only - http://bmjopen.bmj.com/site/about/guidelines.xhtml

658 **List of Abbreviations**

AE	Adverse Event
EDC	Electronic Data Capture
FGS	Female Genital Schistosomiasis
CAA	Circulating anodic antigen
CIN	Cervical intraepithelial neoplasia
ICC	Invasive Cervical Cancer
LLETZ	Large loop excision of the transformation zone
CW	Community workers
HR-HPV	High Risk Human Papilloma Virus
ODK	Open Data Kit
PCR	Polymerase Chain Reaction
RPA	Recombinase Polymerase Amplification
SCW	Schista Community Worker
SRH	Sexual and Reproductive Health
Sh	*Schistosoma haematobium*
SSA	Sub-Saharan Africa
STI	Sexually transmitted infections
Tv	*Trichomonas vaginalis*
VIA	Visual inspection with acetic acid

659

660

For peer review only

For peer review only - http://bmjopen.bmj.com/site/about/guidelines.xhtml

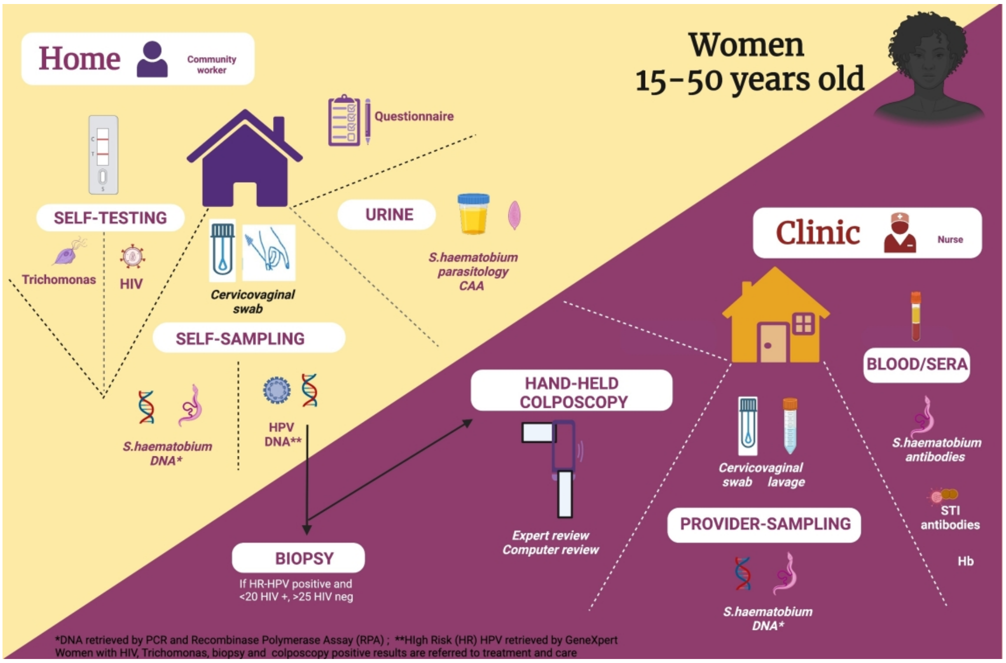

Figure 1. Schista study flow

251x165mm (300 x 300 DPI)

Figure 2 . Flowchart for cervical precancer determination in HIV negative and women living with HIV

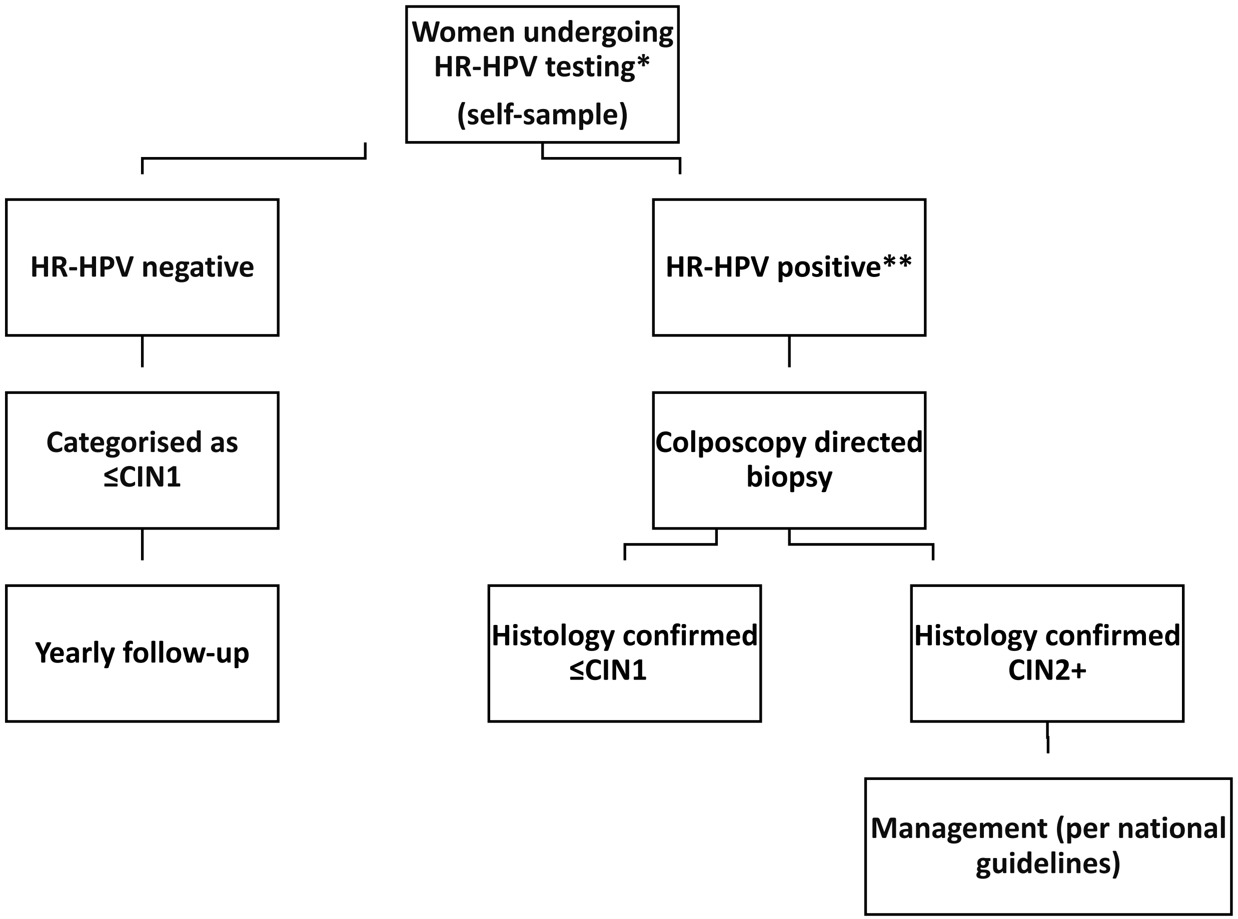

**HR-HPV detected using Xpert; **HR-HPV positive WLHIV aged 20 years and older and HIV-negative women aged 25 years and older only.*

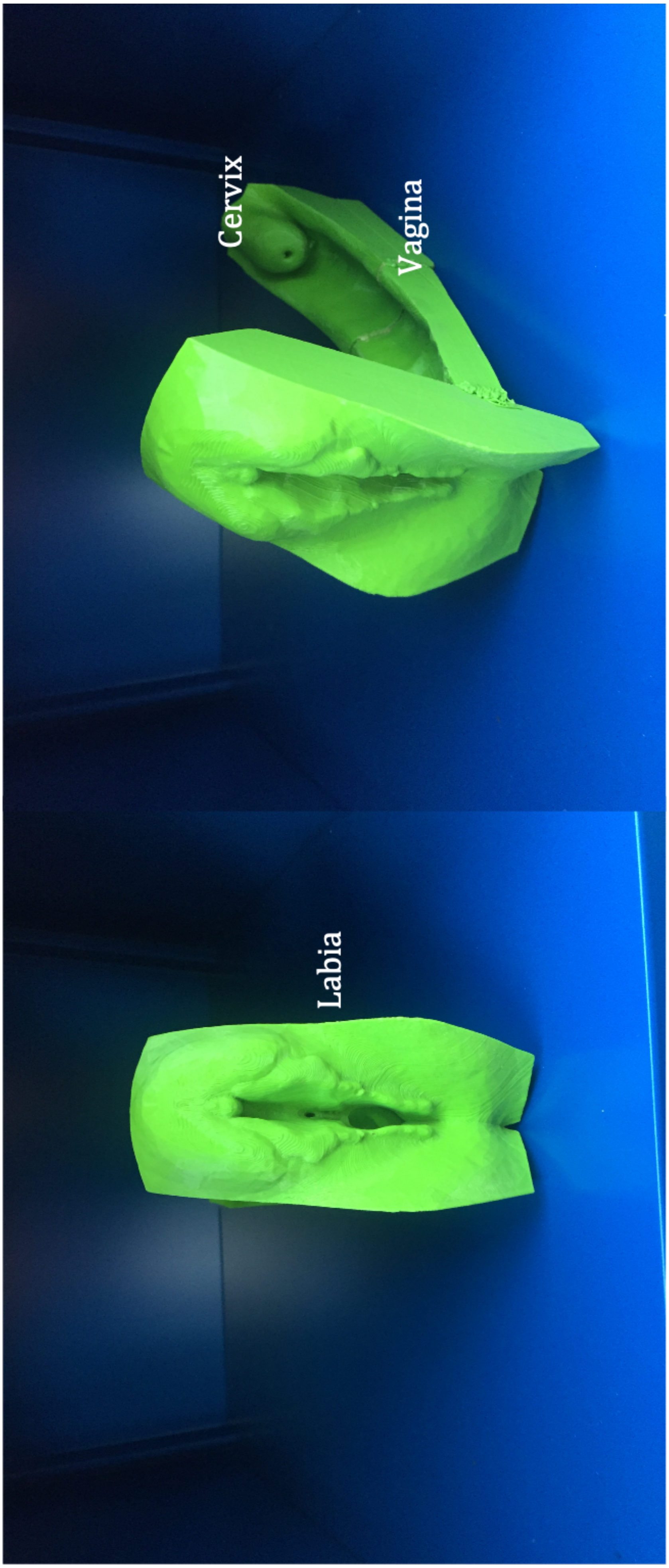

Supplementary figure 1: 3 D models for the teaching of self-sampling
